# Strong population structure deduced from genetics, otolith chemistry and parasite abundances explains vulnerability to localized fishery collapse in a large Sciaenid fish, *Protonibea diacanthus*

**DOI:** 10.1111/eva.12499

**Published:** 2017-07-12

**Authors:** Laura Taillebois, Diane P. Barton, David A. Crook, Thor Saunders, Jonathan Taylor, Mark Hearnden, Richard J. Saunders, Stephen J. Newman, Michael J. Travers, David J. Welch, Alan Greig, Christine Dudgeon, Safia Maher, Jennifer R. Ovenden

**Affiliations:** ^1^ Research Institute for the Environment and Livelihoods Charles Darwin University Darwin NT Australia; ^2^ North Australia Marine Research Alliance Arafura Timor Research Facility Brinkin NT Australia; ^3^ Department of Primary Industry and Resources Northern Territory Government Berrimah NT Australia; ^4^ Centre for Sustainable Tropical Fisheries and Aquaculture James Cook University Douglas QLD Australia; ^5^ Animal Science Queensland Department of Agriculture and Fisheries Brisbane QLD Australia; ^6^ Western Australian Fisheries and Marine Research Laboratories Department of Fisheries Government of Western Australia North Beach WA Australia; ^7^ C_2_O Fisheries Cairns QLD Australia; ^8^ School of Earth Sciences The University of Melbourne Melbourne VIC Australia; ^9^ Molecular Fisheries Laboratory School of Biomedical Sciences The University of Queensland St. Lucia QLD Australia

**Keywords:** croaker, fisheries management, otolith chemistry, parasites, population genetics, stock discrimination

## Abstract

As pressure on coastal marine resources is increasing globally, the need to quantitatively assess vulnerable fish stocks is crucial in order to avoid the ecological consequences of stock depletions. Species of Sciaenidae (croakers, drums) are important components of tropical and temperate fisheries and are especially vulnerable to exploitation. The black‐spotted croaker, *Protonibea diacanthus*, is the only large sciaenid in coastal waters of northern Australia where it is targeted by commercial, recreational and indigenous fishers due to its food value and predictable aggregating behaviour. Localized declines in the abundance of this species have been observed, highlighting the urgent requirement by managers for information on fine‐ and broad‐scale population connectivity. This study examined the population structure of *P. diacanthus* across north‐western Australia using three complementary methods: genetic variation in microsatellite markers, otolith elemental composition and parasite assemblage composition. The genetic analyses demonstrated that there were at least five genetically distinct populations across the study region, with gene flow most likely restricted by inshore biogeographic barriers such as the Dampier Peninsula. The otolith chemistry and parasite analyses also revealed strong spatial variation among locations within broad‐scale regions, suggesting fine‐scale location fidelity within the lifetimes of individual fish. The complementarity of the three techniques elucidated patterns of connectivity over a range of spatial and temporal scales. We conclude that fisheries stock assessments and management are required at fine scales (100 s of km) to account for the restricted exchange among populations (stocks) and to prevent localized extirpations of this species. Realistic management arrangements may involve the successive closure and opening of fishing areas to reduce fishing pressure.

## 1 INTRODUCTION

Nearshore coastal ecosystems provide an important source of food for human populations, supporting 90% of the global wild fish harvest whilst accounting for only 7% of the ocean worldwide (Pauly et al., 2002). As global demand for seafood increases, many exploited species in these regions have declined and large numbers of fisheries are currently fished at unsustainable levels (Jackson et al., 2001; Smith et al., 2010). Sustainable management of exploited fish populations requires detailed knowledge of population structure, natural abundance and the degree of ecologically relevant exchange among harvested stocks (Pauly et al., 2002). The concept of “stocks” as population units has long been anchored in fisheries science and can be defined as groups of fish within a species that are self‐recruiting; share similar growth rates and rates of natural and non‐natural mortality; and may react more or less independently to harvesting (Cadrin, Kerr, & Mariani, 2013).

Methods for delineating stocks have advanced considerably in recent years and include genetic techniques, acoustic telemetry, tagging, otolith chemistry, demographic analysis, otolith shape and meristic data (Hawkins et al., 2016). Genetic approaches speak to both inter‐ and intragenerational timescales as they track the life‐history stages of the fish (i.e., from fertilized eggs to adults) that are interchanged between locations and which subsequently participate in successful spawning. Otolith multi‐elemental signatures and parasite assemblages are ecological markers recording the environment the juvenile and adult fish inhabit and reflect processes occurring within the individuals' lifetime. The integration of multiple techniques that operate over different temporal and spatial scales makes it possible to overcome many of the limitations of single technique approaches and greatly strengthens the inference available from stock structure studies (Abaunza et al., 2008; Begg & Waldman, 1999; Lleonart & Maynou, 2003; Waldman, 1999; Welch et al., 2009, 2015). As an example, Izzo et al. (2017) used an integrated approach to reveal four stocks of the sardine *Sardinops sagax* in Australian waters when it was previously considered a semi‐continuous meta‐population.

Fishes of the family Sciaenidae, commonly known as croakers, are important components of commercial, recreational and indigenous fisheries in tropical and temperate regions worldwide (Lenanton & Potter, 1987). They are targeted for their flesh and, increasingly, for their swim bladders, which are sold fresh or dried in South‐East Asia (Ghosh et al., 2009; Sadovy & Cheung, 2003; Tuuli, 2010). Many species of Sciaenidae have declined in recent decades due to over‐exploitation, and several are now considered threatened. The black‐spotted croaker *Protonibea diacanthus* (Lacapede, 1802) is a large species (>1.5 m max. total length; up to 42 kg mass) that is widely distributed throughout coastal waters and estuaries of the tropical Indo‐West Pacific (Sasaki, 2001). In Australia, it is distributed along the northern coast from Shark Bay, Western Australia to Hervey Bay, Queensland (Bray, 2011). The species grows rapidly and has a maximum recorded age of 13 years (Phelan, 2008). Spawning of *P. diacanthus* in Australia occurs from August to December, and they appear to produce pelagic eggs and have a pelagic larval phase (Froese & Pauly, 2016; Leis & Carson‐Ewart, 2000). Whilst inshore seasonal breeding aggregations of *P. diacanthus* are thought to occur (Welch et al., 2014), direct evidence of the behaviour of the species is limited (Phelan, 2008) and little is known about the size and locations of the aggregations. Commercial and recreational sectors both contribute to total landings (667 tonnes in 2005), but catches have increased recently due primarily to growth in the commercial sector (from 43 to 250 tons per annum between 1995 and 2005; Coleman, 2004; Phelan & Elphick, 2006). Intensive recreational fishing on the east coast of Queensland (Bowtell, 1995, 1998) has also been attributed with reducing fish abundance and size to the point where the catches are almost exclusively limited to immature fish. There is also evidence of over‐exploitation of *P. diacanthus* outside of Australian waters: over‐fishing in India in the 1980s led to local extirpation (James, 1994) and a fishery that once thrived in Hong Kong no longer exists (Sadovy & Cheung, 2003).

Management practices to support the sustainable harvest of *P. diacanthus* in Western Australia, Queensland and the Northern Territory are currently hindered by a lack of knowledge of the species' population structure. In this study, we examined the population structure of the species across north‐western Australia with the expectation of spatially distinct stocks, which would be consistent with the observation of localized depletion. Moreover, we aimed to test the expectation that genetics provides information on a broader spatial scale than otolith chemistry and parasite data in this coastally distributed species. To do so and to demonstrate the power of integrating across techniques, we contrasted population structure results from the three methods and focused on one example where the assignment of individuals to adjacent populations was method‐specific. Finally, we use our results to address the appropriate spatial scale for stock assessment and suggest fisheries management arrangements for *P. diacanthus* across north‐western Australia.

## 2 MATERIAL AND METHODS

### 2.1 Samples

**Figure 1:**
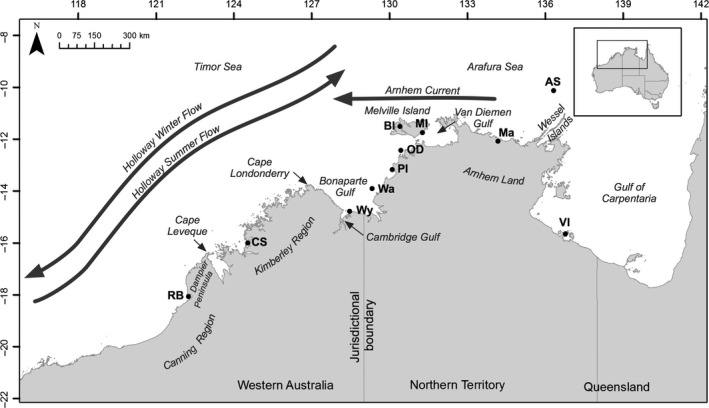
Location of the 11 sampling sites across northern Australia showing the two jurisdictions (Western Australia and Northern Territory). The details for each of the sampling locations are provided in Table 1

A total of 298 fish were collected from 11 sampling locations across north‐western Australia from Roebuck Bay (Western Australia) to the Vanderlin Islands in the Gulf of Carpentaria (Northern Territory) (Figure 1, Table 1). The decline of the species (Phelan, 2002) meant that samples could not be obtained from north‐eastern Australia. The lack of knowledge on the location and size of the aggregation grounds in *P. diacanthus* did not allow the exclusive collection of mature fish on spawning grounds, and thus, fish of different size were included in the analysis. Samples were collected with the assistance of Indigenous marine rangers, fishing tour operators and recreational and commercial fishers. The total length (TL), standard length (SL) and sex of each specimen were recorded. For genetic analyses, muscle tissue or fin clips were sampled and immediately placed into vials containing molecular grade 95% ethanol or 20% dimethyl sulfoxide (DMSO) solution in 5 mol/L NaCl and stored at 4°C in the field and −20°C in the laboratory. The sagittal otoliths were dissected from each fish, cleaned and rinsed thoroughly, dried, and stored in paper envelopes prior to preparation for trace element analysis. Gills and internal body organs were removed, placed into individual bags and frozen until examination for parasites. The same individuals were used for otolith microchemistry, genetic and parasite analyses.

**Table 1:** Details of the 11 sampled locations of *Protonibea diacanthus* across northern Australia

Jurisdiction	Sampling location	Prefix	Sample size	Collection date	Mean TL	Mean age
Western Australia	Roebuck Bay	RB	36	Jul, Aug, Oct, Nov 2014–May, Jun, Jul, Aug 2015	1,018 (720–1,199)	6 (2–10) *(34)*
	Camden Sound	CS	20	Sep, Oct 2013	647 (520–920)	3 (3–4) *(4)*
	Wyndham	Wy	34	May, Jun 2015	1,061 (804–1,300)	5 (3–8) *(26)*
Northern Territory	Wadeye	Wa	25	Jun, Nov 2014	789 (540–1,160)	3 (2–5) *(17)*
	Peron Islands	PI	29	May 2015	N/A	4 (3–5) *(13)*
	Offshore Darwin	OD	17	Oct, Dec 2012–Jul, Sep 2013–Mar 2014	608 (395–1,150)	3 (2–4) *(8)*
	Bathurst Island	BI	28	Nov 2013–Sep, Nov 2014–Aug, Sep 2015	981 (387–1,235)	6 (4–8) *(19)*
	Melville Island	MI	30	Aug 2012–Sep, 2013–Apr, Aug 2015	646 (405–1,170)	2 (2–5) *(26)*
	Maningrida	Ma	30	Aug 2014–Jun, Jul 2015	746 (420–1,210)	3 (2–5) *(17)*
	Arafura Sea	AS	20	Jul 2013	N/A	2 (2–3) *(10)*
	Vanderlin Islands	VI	29	Feb 2014	592 (440–770)	2 (2–3) *(25)*

Mean total length (TL) is expressed in mm and age in years. Range of length and age are indicated in brackets after the mean. Number of fish aged is italicized in brackets after the range.

### 2.2 Data analysis

Population structure was tested in an hierarchical fashion, with genetics being deployed at the broad scale to guide population structure and ecological markers used at the finer scale. The nature of otolith microchemistry reflects fine‐scale spatial variation between environments and is not useful for broad‐scale variation. Indeed, lack of significance (i.e., otolith similarity) on a broad spatial scale does not indicate ecological population homogeneity. Fine‐scale genetic analyses were used to integrate across the three methods for a pair of locations in NW Australia for which one presented a striking genetic heterogeneity.

Genomic DNA from all samples for genotyping was extracted using ISOLATE II Genomic DNA Kit (Bioline) following the manufacturer's instructions to produce 100 μl of eluted DNA from each sample. All the DNA extracts were quantified using the Qubit v3 (ThermoFisher) fluorometric machine. Eleven species‐specific microsatellite loci (*Prd012*,* Prd023*,* Prd044*,* Prd042*,* Prd018*,* Prd045*,* Prd046*,* Prd020*,* Prd036*,* Prd049*,* Prd024*) were genotyped across all the samples as part of multiplexes as described in Taillebois et al. (2016).

The potential for null alleles, large allele dropout and stuttering to interfere with scoring accuracy was checked for each microsatellite locus in each sample using MICROCHECKER version 2.2.3 (Van Oosterhout, Hutchinson, Wills, & Shipley, 2004). Summary statistics for microsatellite loci, including the number of alleles, allelic richness, expected and observed heterozygosity and fixation indexes, were obtained for each sampling location using GENALEX version 6.502 (Peakall & Smouse, 2006). Tests of conformance of genotypic proportions to Hardy‐Weinberg equilibrium expectations were carried out for each locus at each sample location, and tests of genotypic equilibrium between pairs of microsatellites (linkage disequilibrium) were carried out for each sample location, using an exact probability test as implemented in GENEPOP version 4.5 (Rousset, 2008). The exact test was estimated using a Markov Chain that employed 1,000 dememorizations, 500 batches and 1,000 iterations per batch. We accounted for the linkage disequilibrium multiple testing by applying a Bonferroni correction to the *p*‐value. A test with a *p*‐value <.0045 (0.05/number of loci considered in each multiple test) was required to be considered significant.

In order to assess whether locations could be treated as independent genetically cohesive groups on a broad spatial scale, and to test for the extent of admixture within the groups, two contrasting clustering approaches were used. Population assignment and clustering was performed using the Bayesian model‐based clustering program STRUCTURE version 2.3.4 (Pritchard, Wen, & Falush, 2003). For comparative purposes on the broad spatial scale, a discriminant analysis of principal components (DAPC, Jombart, Devillard, & Balloux, 2010) available in the *adegenet* package (Jombart, 2008) for R version 3.3.1 (R Core Team, 2017) was also used. An hierarchical approach was subsequently taken to explore potential substructure within the groups revealed by the STRUCTURE broad‐scale analysis. The hierarchical analyses were also performed using STRUCTURE. Methodology for STRUCTURE and DAPC is detailed in Appendix A1 in Appendix S1.

To test alternate *post hoc* observations of genetic structure revealed by our different approaches, we assessed the per cent of genetic variance explained by the groupings deduced from STRUCTURE and DAPC results using an analysis of molecular variance (AMOVA) as implemented in ARLEQUIN. The significance of any differentiation was determined by permutation of 22,000 replicates.

Pairwise *F*_ST_ values were calculated (i) to further investigate the population structure within the groups revealed with the clustering approaches and (ii) to test whether *P. diacanthus* is constrained by a pattern of isolation by distance (IBD). Fixation indices (*F*_ST_) between pairs of sample localities were estimated as implemented in ARLEQUIN version 3.5.2.2 (Excoffier & Lischer, 2010). We performed a Mantel test of *F*_ST_/(1 − *F*_ST_) (G) versus geographic (D) distances among locations using ARLEQUIN.

To investigate the fine‐scale population structure and provide information on the dispersal of fish, the microchemistry of the otolith was examined in two sections of the otoliths: the near core and the margin. The near core area is located just outside the first opaque zone ~500 μm from the core of the otolith and represents the juvenile phase; and the otolith margin (edge) was sampled adjacent to the *sulcus acusticus* to represent growth in the period prior to capture. A total of 11 trace elements (^7^Li, ^25^Mg, ^23^Al, ^49^Ti, ^53^Cr, ^55^Mn, ^60^Ni, ^63^Cu, ^66^Zn, ^88^Sr, ^138^Ba) and the internal standard (^43^Ca) were analysed from the two ablation zones in each otolith. Details on the general methodology for the otolith preparation, the laser ablation and data transformation are presented in Appendix A2 in Appendix S1. For all elements, the ratio of element isotope intensity to Ca intensity was used to estimate the element:^43^Ca ratio. These ratios were converted to molar ratios and were expressed as element:Ca molar ratios in mmol/mol or μmol/mol (Appendix B1 in Appendix S2).

Parasites were also used as biological tags to investigate the fine‐scale population structure of *P. diacanthus*. Direct life cycle parasites generally have short‐lived larval stages that are unable to cover large distances and should reflect locality of origin. Indirect life cycle parasites, on the other hand, can travel through multiple hosts and thus have a distribution pattern that is ultimately at the mercy of a number of different factors. Actual life cycles of the majority of aquatic parasites remain unknown, and we can only generalize as to hosts within the cycle based on current knowledge. Initially, analyses were performed for external parasites (=direct) and larval stages (=indirect or permanent parasites) separately. Both analyses showed significant separation among sites. We subsequently combined the data for direct and indirect parasites for the final analyses, as this provided more robust and biologically reliable and interpretable results (see below).

Details on the general methodology for the parasite extraction and examination are presented in Appendix A3 in Appendix S1 Summary statistics of the parasites data were compiled for each location, as well as for all fish examined and are presented in Appendix B2 in Appendix S2. This included mean abundance (total number of individuals of a particular parasite per collection location divided by the total number of fish from that location examined, including uninfected hosts) and prevalence (number of hosts infected with a particular parasite divided by the number of hosts examined, expressed as a percentage) for each of the parasite species deemed suitable for use in the analyses, following the terminology of Bush, Lafferty, Lotz, and Shostak (1997). Only parasites with a prevalence greater than or equal to 10% in at least one of the locations were used in the analysis (Bush, Aho, & Kennedy, 1990); additional parasites were removed from analyses if they could not be easily identified and/or accurately counted (MacKenzie & Abaunza, 1998).

The otolith and parasite data were broken down into three geographic regions for statistical analyses (i) because these ecological markers are thought to operate over smaller spatial and temporal scales compared to the genetics; hence, they require a smaller scale of analysis and (ii) to test of the current jurisdictional management boundary between Western Australia and the Northern Territory and the regional management units across the Northern Territory. Each region included the geographically closest sample location from the adjacent region to allow inter‐region comparisons. The Western region includes all of the Western Australian locations (RB, CS and Wy) as well as Wa, which is the closest Northern Territory sample location (Figure 1). The Darwin region includes all the Northern Territory locations from Wa to MI (Wa, PI, OD, BI and MI). The Arnhem/Gulf region includes all the Arnhem Land and Gulf of Carpentaria populations of the Northern Territory (Ma, AS and VI) as well as MI, which is the closest Northern Territory sample location.

All data analysis was performed using R (R Core Team, 2017). Pairwise distributions of all variables used in each analysis for the parasite and otolith data sets were inspected for extreme outliers, unsatisfactory covariate distributions, and the presence of collinearity. Where necessary abundance data were log(*Y* + 1) transformed. Homogeneity of the covariance matrices for the grouping factor was assessed using Box's *M*‐test based on the chi‐square approximation with the *biotools* package in R (da Silva, 2016).

Fish are known to accumulate longer‐lived parasite species with age (Rohde, 1982), which will affect differences in parasite fauna if samples contain individuals of different ages. All parasite species that were selected on prevalence were examined for their correlation with host length (as an indicator of age) using linear models. Where length was significantly correlated with abundance, numbers greater than zero were adjusted to the mean host size (total length) using the methods described in Moore, Buckworth, Moss, and Lester (2003).

Spatial variation in parasite assemblages and the otolith near core and margin chemistry among regions and locations within regions were investigated separately using single‐factor multivariate analysis of variance (MANOVA). Linear discriminant function analysis (LDFA) was conducted to provide a statistical and visual indication of the similarities of either the parasite communities or the multi‐elemental otolith microchemical signatures among samples at the regional spatial scale using the *MASS* package in R (Venables & Ripley, 2002). LDFA classification success rates and an associated proportional chance criterion (the expected proportion of correct classification by chance alone) were calculated (Poulin & Kamiya, 2015) for each location within the regions. To show the separation achieved for the groups in the analysis, the first two discriminant functions for each individual were plotted and the 95% confidence ellipses around the centroid means of the first two discriminant functions for each group in the sample using the *ellipse* package in R (Murdoch & Chow, 2013).

To test the effect of distance between locations on the parasite assemblages, and the otolith elemental concentrations in the near core and the margin, we performed a Mantel test of the Jaccard dissimilarity using the mean differences in number and concentrations, respectively, between each pair of locations versus geographic distances among locations using the *vegan* package in R (Oksanen et al., 2017).

The individual assignment of fish was tested through a Bayesian approach across all fish for otoliths and parasites. Classes and posterior probabilities were calculated by jackknife cross‐validation to assign individuals to most probable locations within each region. In a couple of locations where the genetic structure gave evidence of genetic heterogeneity within locations, STRUCTURE was used to calculate posterior probabilities and assign individuals to most probable locations. Individual mis‐assignments were compared across techniques to test whether each technique provided different information or not (i.e., see whether the same fish were mis‐assigned to the same locations between techniques).

## 3 RESULTS

Genotypes from 11 microsatellite loci were obtained for 284 individuals of *P. diacanthus*. The level of missing data was low (2.15%) and as such was retained and identified as missing data in the subsequent analyses (missing data were calculated as the number of genotypes that could not be scored over the total number of genotypes present in the ideal data set, 11 × 284). The number of alleles per locus ranged from 5 (*Prd046*) to 21 (*Prd012*) (Appendix B3 in Appendix S2). The data were free of scoring errors as evaluated by MICROCHECKER. Genotypes were rescored and corrected until there was no evidence of null alleles at these or other locations and loci. Deviations from Hardy‐Weinberg proportions (HWP) were detected 14 times (*p*‐value <.05 for *Prd044* at RB, *Prd023* at RB, Wa and BI, *Prd042* at OD, *Prd012* at Wa and Ma, *Prd018* at CS and Ma, *Prd020* at PI, OD and AS, *Prd036* at RB and *Prd024* at PI) out of the 121 tests performed for each locus against each location. There was no significant test for linkage disequilibrium between pairs of loci across all locations after Bonferroni correction (*p*‐value <.0045). The analysis proceeded without removing loci or individuals from the data set. Heterozygosity was moderate to high for all loci across all locations (0.659 ± 0.179, Appendix B3 in Appendix S2) and generally similar to expectations for marine fish (approximately 0.7, DeWoody & Avise, 2000).

**Figure 2:**
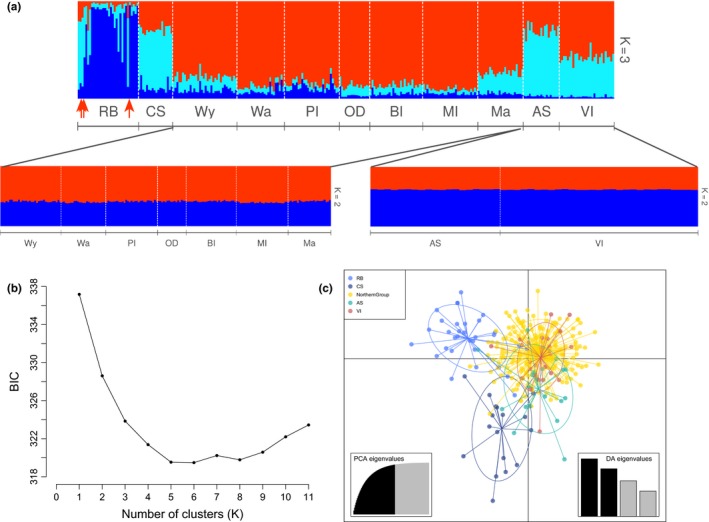
Summary of Bayesian model‐based and discriminant analysis of principal components (DAPC) approaches for population clustering of microsatellite data from *Protonibea diacanthus*. (a) Plot of the Bayesian model‐based population clustering using STRUCTURE for *K* = 3 genetic clusters across the entire data set, for *K* = 2 within the northern group (Wy‐Wa‐PI‐OD‐BI‐MI‐Ma) and the gulf locations (AS‐VI). Each vertical line represents an individual and the posterior probability proportions of its genotype assigned to the different genetic clusters. Individuals are plotted from west to east along the sampling gradient, and populations are abbreviated following Table 1. Red arrows point at the individuals from RB that are assigned to CS. The admixture model and population information were used in the analysis. (b) Changes in mean Bayesian Information Criterion (BIC) values in successive *K*‐means clustering. (c) Ordination plot of the DAPC for the five predefined clusters (RB, CS, Wy‐Wa‐PI‐OD‐BI‐MI‐Ma = Northern Group, VI and AS). Individual genotypes appear as dots, and locations are depicted by colours and 95% inertia ellipses. The bottom‐left inset shows the number of principal component retained and the cumulative variance they explain; and the bottom‐right inset shows the eigenvalues for the four principal components in relative magnitude with *x*‐axis and *y*‐axis constituting the first two principle components, respectively

Four distinct populations were identified using Bayesian model‐based clustering methods. The optimum number of genetic clusters returned by ∆*K* in STRUCTURE was 5 without *LOCPRIOR* and 3 with *LOCPRIOR*. The *LOCPRIOR* parameter enhanced the population structure pattern (Appendix B4 in Appendix S2); hence, the following results describe the observed pattern using location information as a prior. For *K* = 3, four geographically distinct populations or groups of populations were revealed. The western populations RB and CS were distinct from each other, and a group of central northern populations Wy‐Wa‐PI‐OD‐BI‐MI‐Ma appeared to be similar. There was also an eastern group with locations AS and VI appearing similar to each other. Despite the similarity between AS‐VI and CS genotypes when *K* = 3, they were assumed to be different populations or group of populations because of their geographic distance (Figure 2a). Independent investigations of hierarchical structure within the northern group Wy‐Wa‐PI‐OD‐BI‐MI‐Ma and within the Gulf locations AS‐VI did not reveal further substructure within those two groups (Figure 2a). Overall, the groups favoured by STRUCTURE were the following four: RB, CS, Wy‐Wa‐PI‐OD‐BI‐MI‐Ma, AS‐VI.

In successive *K*‐means clustering using *find.clusters* in R, the Bayesian Information Criterion (BIC) showed an initial sharp decline in values until *K* = 5 after which BIC values increased (Figure 2b). To match the number of genetic clusters revealed by *find.clusters*, the individuals were partitioned into five groups of locations that would take into account the genetic structure previously highlighted with STRUCTURE. The Gulf of Carpentaria locations AS and VI were here split based on their geographic distance. The five groups used as a prior in the DAPC were as follows: RB, CS, Wy‐Wa‐PI‐OD‐BI‐MI‐Ma, AS and VI. The optimal number of PCs with the highest mean successful assignment and the lowest mean squared error was 30. When partitioning the individuals into five groups, DAPC revealed overlapping distributions of geographic groups on the ordination plot that indicated a low degree of genetic differentiation between these groups or locations. The DAPC separated RB from other locations along the first principal component axis (eigenvalue = 47.99) (Figure 2c). Along the second component axis (eigenvalue = 35.59), AS was plotted distant from the Western Australia locations RB and CS as well as the Wy‐Wa‐PI‐OD‐BI‐MI‐Ma group but closer to VI (Figure 2c).

AMOVA was used to compare the different scenarios supported by STRUCTURE and DAPC. The 4‐ and 5‐group scenario highlighted by STRUCTURE and DAPC, respectively, explained 2.15% (*p*‐value = .000) and 2.28% (*p*‐value = .000) of the total genetic variation. As both scenarios explained very similar among group genetic variation, they are both likely to represent biological population structure. However, the 4‐group scenario revealed by STRUCTURE will be used in the discussion, as it is the result of unsupervised analyses, which was not the case for DAPC.

The pairwise *F*_ST_ values confirmed some of the results found with the clustering approaches. A pattern of genetic differentiation with low but significant population‐pairwise *F*_ST_ values (range 0.009–0.054, Table 2) was observed, and the overall *F*_ST_ value was 0.014. The pattern revealed that two locations in the western part of the sampling area (RB and CS) and two locations in the eastern part of the sampling area (AS and VI) were genetically distinct from almost all other locations surveyed in the study. By contrast, the seven locations (Wy, Wa, PI, OD, BI MI, Ma) in between the eastern and western locations formed an undifferentiated group where only three of 42 pairwise *F*_ST_ values were significant and none of them were significant after Bonferroni correction.

**Table 2:** Pairwise *F*_ST_ estimates based on 11 microsatellite data from 284 individuals of *Protonibea diancanthus* among (a) 11 sampling locations

	RB	CS	Wy	Wa	PI	OD	BI	MI	Ma	AS	VI
RB		**0.000**	**0.000**	**0.000**	**0.000**	**0.001**	**0.000**	**0.000**	**0.000**	**0.000**	**0.000**
CS	**0.033**		0.096	0.089	**0.000**	0.064	0.014	0.006	0.008	**0.001**	0.012
Wy	**0.029**	0.006		0.542	0.008	0.974	0.008	0.817	0.532	**0.000**	**0.000**
Wa	**0.033**	0.009	−0.001		0.385	0.991	0.743	0.984	0.806	**0.002**	0.069
PI	**0.035**	**0.029**	0.009	0.000		0.544	0.009	0.229	0.265	**0.000**	**0.000**
OD	**0.030**	0.014	−0.009	−0.012	−0.002		0.822	0.984	0.975	0.138	0.515
BI	**0.035**	0.016	0.010	−0.003	0.011	−0.005		0.574	0.091	**0.000**	0.007
MI	**0.033**	0.014	−0.004	−0.009	0.001	−0.012	−0.002		0.695	**0.001**	0.095
Ma	**0.034**	0.019	−0.001	−0.004	0.002	−0.011	0.007	−0.004		0.039	0.044
AS	**0.054**	**0.021**	**0.022**	**0.018**	**0.030**	0.007	**0.031**	**0.017**	0.011		0.020
VI	**0.048**	0.014	**0.015**	0.007	**0.016**	−0.001	0.013	0.004	0.009	0.011	

Lower diagonal, *F*_ST_ estimates; upper diagonal, *p*‐values of the corresponding *F*_ST_ estimate, the comparisons that differed significantly from zero (*p*<.05) are shaded in grey, the ones that differed after Bonferroni correction (*p*<.0045) are in bold. Location prefixes follow Table 1.

**Figure 3:**
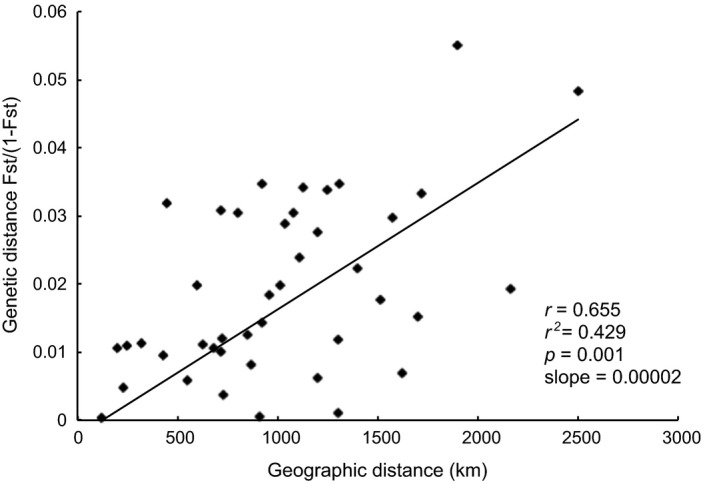
Isolation‐by‐distance analysis generated from 1,000 Mantel randomizations. Genetic distance *F*_ST_/(1 − *F*_ST_) against geographic distance (km) and corresponding values

To evaluate the spatial processes that drive population structure, an isolation‐by‐distance model was examined. The Mantel correlation coefficient *r* between the genetic and geographic distance matrices was equal to 0.655 (coefficient of determination *r* ^*2 *^= .429) and was statistically significant (*p*‐value = .001). There was a linear relationship between *F*_ST_/(1 − *F*_ST_) and geographic distances (Figure 3). Thus, fish from nearby locations tended to be genetically more similar than expected by chance and genetic differences increased linearly with distance. There was no correlation between the mean elemental otolith composition and the geographic distance between locations (near core *r* = −.225, *p*‐value = .85; margin *r* = .449, *p*‐value = .05). The correlation coefficient between the parasites abundance and the geographic distance between locations was low and marginally significant (*r* = .434, *p*‐value = .04).

Ecological markers (i.e., otolith microchemistry and parasite abundances) provided further information about population structure in *P. diacanthus* and information on the individuals' life history. Of the 11 elements measured, concentrations of ^7^Li, ^25^Mg, ^55^Mn, ^60^Ni, ^63^Cu, ^66^Zn, ^88^Sr and ^138^Ba in the near cores and margins of *P. diacanthus* otoliths were measured above the LOD in a total of 286 fish (Appendix B1 in Appendix S2). A total of 291 *P. diacanthus* were examined for the parasitology component of this study. Overall, all fish were infected with at least one parasite individual and a total of 44 different parasites species were identified (Appendix B2 in Appendix S2). Of these, 11 were excluded from the analyses based on prevalence and a further four groups (e.g., Anisakid nematode larvae, Ancyrocephaline and Diplectanid monogeneans) were excluded based on issues with identification and/or enumeration. The mean parasite species richness was 5.8 (range 1–12) per host, and the mean abundance 57.3 (range 1–653) parasite individuals per host.

**Figure 4:**
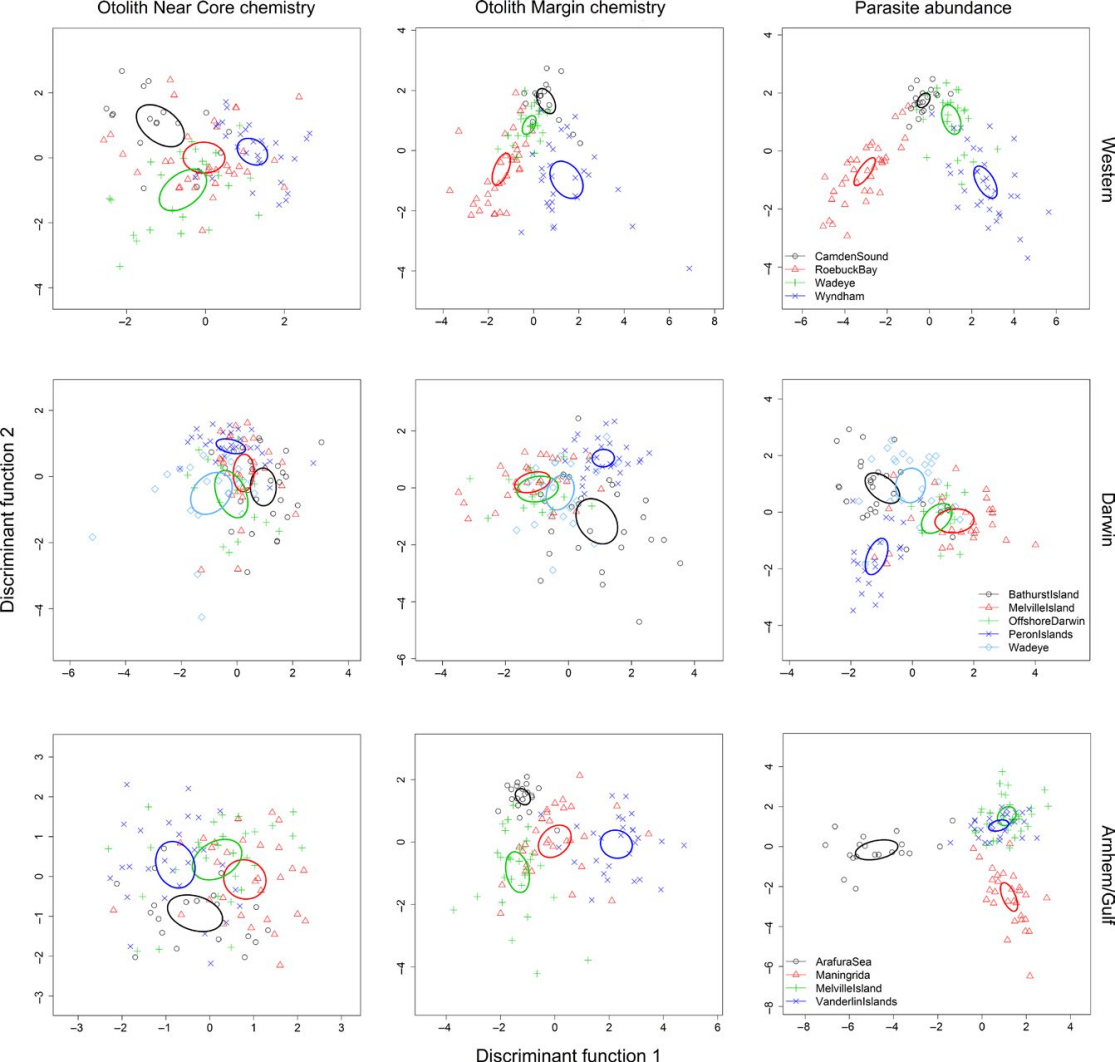
Plots of the first two discriminant function scores showing spatial variation in the parasite assemblage and the multi‐elemental otolith near core and margin signatures of *Protonibea diacanthus* collected from 11 locations within three regions: Western region, Darwin region and Arnhem/Gulf region. Ellipses are 95% confidence intervals around the group centroid for each location within each region, and data points represent individual fish

The multi‐elemental signatures of the near core and margins and the parasite abundances differed significantly among the three broad‐scale regions (Western, Darwin, Arnhem/Gulf) and among locations within each of the three regions (MANOVA, all tests *p*‐value <.001, Table 3, Figure 4). Average classification success in the LDFA among all locations was 54% for the otolith margin, 31% for the otolith near core and 67% for the parasites (Table 4) and was much higher than expected by chance (9% for the otolith and 10% for the parasites), suggesting a nonrandom distribution of the otolith microchemistry and parasite abundance variability across the sampled range. Classification success for both margin (71% for the Western region, 57% for the Darwin region and 74% for the Arnhem/Gulf region) and near core (57% for the Western region, 44% for the Darwin region and 38% for the Arnhem/Gulf region) was higher when the data for each region were analysed separately, but the % classification success relative to the % expected by chance was higher when all sites were analysed simultaneously (Table 4, Figure 4). Similar to the otolith chemistry analysis, separation of the analyses into regions increased reclassification success in parasite abundances, with 81% for the Western region, 56% for the Darwin region and 89% for the Arnhem/Gulf region (Table 4, Figure 4). For both otoliths and parasites, the classification success was greater for the Arnhem Gulf and Western regions than for the Darwin region.

**Table 3:** Results of the multivariate analysis of variance (MANOVA) investigating the spatial variability in parasite assemblage and otolith near core and margin microchemistry of *Protonibea diacanthus* among and within each region

Source	Source, error *df*		Pillai's trace			*F*		
	Otolith	Parasite	Margin	Near Core	Parasite	Margin	Near Core	Parasite
Among region	80, 2, 200	10, 278	1.80	1.04	4.24	7.973^a^	4.101^a^	6.58^a^
Among locations within the Western region	24, 291	3, 111	1.11	0.88	2.07	7.120^a^	5.006^a^	8.748^a^
Among locations within the Darwin region	32, 468	4, 117	1.08	0.72	1.61	5.392^a^	3.203^a^	3.03^a^
Among locations within the Arnhem/Gulf region	24, 294	3, 103	1.27	0.59	2.28	8.992^a^	2.997^a^	12.807^a^

*df* is the degree of freedom.

a Indicates a *p*<.001.

**Table 4:** Jackknife reclassification success of the linear discriminant function analysis (DFA) for the overall otolith near core and margin chemistry and parasite assemblage of *Protonibea diacanthus*

	*n*	Margin	Near core	Parasites
		% Correct	% Correct	% Correct
(a) Among regions				
All Locations	286	54 (9)	31 (9)	67 (10)
(b) Within the Western region				
Roebuck Bay	34	76	50	86
Camden Sound	18	67	61	95
Wyndham	30	77	73	68
Wadeye	24	58	42	80
Total	106	71 (26)	57 (26)	81 (26)
(c) Within the Darwin region				
Wadeye	24	33	38	56
Peron Islands	29	93	62	73
Offshore Darwin	17	12	18	65
Bathurst Island	27	63	48	43
Melville Island	29	62	41	50
Total	126	57 (21)	44 (21)	56 (21)
(d) Within the Arnhem/Gulf region				
Melville Island	29	79	41	80
Maningrida	30	47	40	90
Arafura Sea	20	95	25	90
Vanderlin Islands	28	82	43	97
Total	107	74 (26)	38 (26)	89 (26)

Data are presented as the % of individuals that reclassify to their collection location. Poulin and Kamiya's (2015) proportional chance criterion is shown in bracket after the total classification success within each region.

Classification success was higher for the margin than the near core across all locations (except Wa and OD in the Darwin region), reflecting the fact that the margin sample incorporated growth at the sampling location immediately prior to collection (i.e*.,* samples collected from the same location in the same year). In contrast, the near core (juvenile phase) samples incorporated growth from different years because fish in each location were of variable ages and sources may vary by age and cohort. Individual mis‐assignments were compared between near core and margin, and there was no evidence of matching pattern of mis‐assignment (Appendix B5 in Appendix S2). Fish that were mis‐assigned using the near core were not necessarily mis‐assigned using the margin; and if they were, the assigned location was not necessarily the same. Classification success was generally higher for the parasites than the otolith except BI and Wy where both margins and near cores had higher classification rates than parasites; and PI and AS where only the margins had higher classification rates than the parasites. Individual mis‐assignments were compared between parasites and margin, and there was no evidence of matching pattern of mis‐assignment (Appendix B5 in Appendix S2). Fish that were mis‐assigned using the parasites were not necessarily mis‐assigned using the margin, and if they were, the assigned location was not necessarily the same.

The results from the genetic STRUCTURE analyses showed that RB is a heterogeneous collection of genotypes compared to all other locations with individuals presenting matching genotype proportions to individuals from CS location (Figure 2a). Further full Bayesian assignment tests between RB and CS revealed that individuals having outlier genetic signature did not necessarily have distinct otolith or parasite characteristics. Of the three individuals that were mis‐assigned with the genetics, fish 3167 and 3540 were also mis‐assigned using the near core and the parasites, respectively (Table 5). Assuming parasites and otolith margins provide information about postlarval development; and near core and genetic provide information about the location of early stage development, we tested the hypotheses that otolith near core would correspond to the genetics; and that parasites and otolith margin would provide similar assignments too. Off the eight fish that were mis‐assigned using the near core, only one was similarly mis‐assigned for the genetics (fish 3167). Of the four fish that were mis‐assigned for the margin and the two for the parasites, only one was mis‐assigned for both parasites and margin (fish 3518).

**Table 5:** Assignments of *Protonibea diacanthus* individuals between Roebuck Bay (RB) and Camden Sound (CS) locations based on a Bayesian approach for the genetics, otolith near core and margin and parasites

Sample	Size (mm)	Sampling location	Genetics	Near Core	Margin	Parasites
			Assigned location	Assigned location	Assigned location	Assigned location
2504	623	CS	CS	RB	CS	CS
2505	580	CS	CS	CS	CS	RB
2506	827	CS	CS	RB	CS	CS
2508	594	CS	CS	CS	CS	CS
2509	638	CS	CS	CS	CS	CS
2510	524	CS	CS	CS	CS	CS
2511	539	CS	CS	CS	CS	RB
2512	920	CS	CS	RB	CS	CS
2513	905	CS	CS	CS	CS	RB
2514	595	CS	CS	CS	CS	CS
2516	565	CS	CS	CS	CS	CS
2517	631	CS	CS	CS	CS	CS
2518	649	CS	CS	RB	CS	CS
2519	671	CS	CS	RB	CS	CS
2520	601	CS	CS	RB	CS	CS
2521	634	CS	CS	RB	CS	CS
2522	673	CS	CS	CS	CS	CS
2523	520	CS	CS	CS	CS	CS
2507	650	CS	NA	NA	NA	CS
2515	595	CS	NA	NA	NA	CS
3518	720	RB	RB	RB	CS	CS
3539	1,114	RB	RB	CS	CS	RB
3540	1,140	RB	CS	RB	CS	RB
3545	1,019	RB	RB	RB	CS	RB
3521	1,057	RB	RB	NA	NA	RB
3523	835	RB	RB	NA	NA	RB
3530	1,030	RB	RB	RB	NA	RB
3167	1,040	RB	CS	CS	RB	RB
3168	1,009	RB	RB	RB	RB	RB
3169	1,005	RB	CS	RB	RB	RB
3170	1,010	RB	RB	CS	RB	RB
3171	1,055	RB	NA	RB	RB	RB
3172	975	RB	NA	RB	RB	RB
3173	1,100	RB	NA	RB	RB	RB
3174	1,000	RB	NA	RB	RB	RB
3519	910	RB	RB	RB	RB	RB
3520	1,050	RB	RB	RB	RB	RB
3522	1,104	RB	RB	RB	RB	RB
3524	892	RB	RB	RB	RB	RB
3525	1,035	RB	RB	CS	RB	RB
3526	1,090	RB	RB	RB	RB	RB
3527	1,135	RB	RB	RB	RB	RB
3528	1,136	RB	RB	RB	RB	RB
3529	810	RB	RB	RB	RB	CS
3531	1,095	RB	RB	RB	RB	RB
3532	1,100	RB	RB	RB	RB	RB
3533	1,182	RB	RB	RB	RB	RB
3534	899	RB	RB	RB	RB	RB
3535	980	RB	RB	CS	RB	RB
3536	930	RB	RB	RB	RB	RB
3537	988	RB	RB	RB	RB	RB
3538	1,199	RB	RB	CS	RB	RB
3541	984	RB	RB	RB	RB	RB
3542	1,012	RB	RB	CS	RB	RB
3543	870	RB	RB	RB	RB	RB
3544	1,152	RB	RB	CS	RB	RB

## 4 DISCUSSION

In this study, the combined application of genetic markers, otolith chemistry and parasites across the same specimens provided three independent lines of evidence and considerable power to detect the highly complex population structure of *P. diacanthus* across northern Australia. Relatively few studies to date have used this integrated approach to investigate population structure and connectivity or for stock identification (Abuanza et al., 2008; Welch et al., 2015; Izzo et al., 2017). These studies also concluded that the integrated use of multiple techniques considerably improved confidence in stock identification using index of stock differences (ISD) thresholds definition (Izzo et al., 2017; Welch et al., 2015) and were particularly effective for species with broadly dispersing larvae or adults that exhibit little or no genetic structure across their range (Waldman, 1999). As a case in point, our study revealed the existence of major genetic breaks along the north‐western Australian coastline and thus the presence of several discrete breeding populations of *P. diacanthus*. Prior to settlement, genetic results suggest that currents and geographic barriers to dispersal may limit exchange among collection locations. The ecological markers (otoliths and parasites) strongly suggest that *P. diacanthus* is generally sedentary following larval settlement.

### 4.1 Site fidelity and dispersal

Whilst the results of this study suggest high levels of site fidelity after larval settlement for *P. diacanthus* based on the fine‐scale population structure, it should be noted that there was overlap in the multivariate analyses of parasite assemblage compositions and otolith elemental signatures between some locations that potentially reflect dispersal by a proportion of juvenile or adult fish. Similar to the IBD pattern observed in the genetic analyses, the correlation between geographic distance and parasite assemblage diversity showed a decline in the similarity of parasite assemblages with increasing distance. The genetic IBD pattern indicates that reproduction is more likely to occur between individuals in the same location. The positive correlation between distance and parasite assemblage dissimilarity could be interpreted in a similar manner, suggesting that parasites are shared by the dispersal of fish within and among neighbouring populations, which may be responsible for the relative similarity of neighbouring locations. Alternatively, it may be that similarity in parasite assemblages among neighbouring sites is driven by (i) dispersal of parasites independent of *P. diacanthus* movement (e.g., *via* other hosts); (ii) the spatial distributions of the endemic areas of the different parasite species (MacKenzie & Abaunza, 1998); or (iii) habitat preferences of the parasite species, with neighbouring locations having more similar habitat conditions than distant locations (MacKenzie & Abaunza, 1998). Under these three scenarios, dispersal of fish among locations would not be necessary to explain the relationship between parasite assemblage similarity and geographic distance.

In the case of otolith chemistry, overlap in multi‐elemental signatures among locations is likely to be driven by local geochemical characteristics, including the underlying seabed and nearby river catchment geology, as well as physicochemical factors such as water temperature and salinity and their effects on fish physiology and local ecological processes (Campana & Thorrold, 2001; Elsdon & Gillanders, 2002, 2004). Whilst such factors could potentially vary systematically over geographic gradients, we found no evidence of a relationship between multi‐elemental signatures and geographic distance. Indeed, several very distant sites (e.g., CS and MI) showed high levels of overlap in otolith chemical signatures, but it is extremely unlikely that there was any exchange of individual fish between these distant locations given the weight of evidence across the other techniques (parasites and genetics) of location fidelity and limited dispersal. Rather, the misclassification of individuals to distant locations appears to be due to the high number of locations relative to the limited number of elements that contributed to location separation in the discriminant function analysis. Similar overlap and misclassification in otolith chemical signatures among locations have been reported previously both in freshwater (e.g*.,* Macdonald & Crook, 2014) and marine (e.g., Miller, Banks, Gomez‐Uchida, & Shanks, 2005) studies.

As previously mentioned, the generally lower classification success in the near core compared to the margin can, at least in part, be attributed to the fact that the near core samples incorporated growth from different years (i.e*.,* different age cohorts) within each location (see also Gillanders, 2002). It is also possible that movement by fish after the juvenile phase accounts for some the reduced classification success in the near core analyses. Yet despite the potential for variability among fish of different ages within a location, our analyses consistently revealed highly significant spatial differences in otolith chemistry signatures among locations. This finding agrees with our conclusion of location‐level population structure based on parasite assemblages and, further, suggests that at least some spatial structuring is maintained from the early stages of the life history of *P. diacanthus*. Even though ages were available for our samples, the low number of samples per location combined with the wide range of ages resulted in low numbers per cohort. Hence, cohorts could not be compared in this study, and further research is required to determine the extent to which mis‐classification of fish can be attributed to temporal variation in otolith chemical signatures versus other potential causes (e.g., immigration from other locations).

### 4.2 Population structure and connectivity

At broader spatial scales, the genetic analyses revealed the existence of major discontinuities along the north‐western Australian coastline, resulting in several genetically discrete populations of *P. diacanthus*. This finding is based on mixed age samples, and we would predict stronger spatial structuring if mature fish were collected on spawning grounds. Major genetic breaks distinguished populations at RB, CS, a homogenous group of locations in the Northern Territory (from Wy to Ma), the offshore region of the AS and the VI in the Gulf of Carpentaria. The deepest genetic structure observed was between RB in Western Australia and the rest of the sampling range. The distance between RB and the nearest location CS (~400 km) may partly explain this pronounced genetic break. However, similar or greater distances between other locations (e.g*.,* 425 km between Ma and MI or 630 km between the AS and VI) did not result in such genetic discontinuity.

The geomorphology and local hydrodynamic regime of the northern coastline of Western Australia may influence the population genetic structure of *P. diacanthus* across its range. The tidal regime, the composition of the substrate and the extent of the riverine network influence the size of the mangrove communities in this area. Thus, the Kimberley and Canning bioregions separated by the Dampier Peninsula (Figure 1) (Semeniuk, 1996; Thackway & Cresswell, 1998) differ significantly in mangrove characteristics. The tip of the Dampier Peninsula has been identified as an important biogeographic break for marine species (Hutchins, 2001; Travers, Potter, Clarke, Newman, & Hutchins, 2010; Wilson, 2013) and coincide with a pronounced shift in the underlying geology (e.g., from sedimentary sandstone in the north to unconsolidated sand and silt in the south) and associated dominant benthic habitats (e.g., from coral reefs in the north to soft substrate communities in the south). This barrier also coincides with a prominent increase in tidal currents and associated water turbidity to the north. The Kimberley‐Canning border has the largest tropical tidal range (~12 m) and some of the fastest tidal currents in the world (2.5 m/s), and experiences massive volumes of freshwater in a highly turbid plume from the Fitzroy River during the monsoonal wet season (Wolanski & Spagnol, 2003). The discontinuity between the Kimberley and Canning geomorphology (macrotidal, jagged “ria” coastline with extensive mangrove forests *vs* smoother coastline with no rivers) creates contrasted marine coastal habitats, inhibiting larval dispersal and reinforcing spatial population structure. Likewise, quantitative analyses of fish assemblages have revealed two main biogeographic regions, the Kimberley and Canning coasts (Hutchins, 2001; Travers, Newman, & Potter, 2006; Travers, Potter, Clarke, & Newman, 2012; Travers et al., 2010; Wilson, 2013), matching the genetic groups reported here. Our study is the first to identify a clear genetic break between the Kimberley and the Canning bioregions in coastal fish. Across the same broader area, studies on threadfin species (*E. tetradactylum* and *Polydactylus macrochir*) also reported a strong east‐west phylogeographic break between RB and populations of Van Diemen Gulf or in the Gulf of Carpentaria (Horne, Momigliano, Welch, Newman, & van Herwerden, 2011; Horne, Momigliano, Welch, Newman, & Van Herwerden, 2012), but lacked intermediate sampling between these locations that may have identified a Kimberley‐Canning break. Similarly, a genetic study on grey mackerel (*Scomberomorus semifasciatus*) highlighted the distinctiveness between Western Australia (Pilbara region to the south of the Canning) and the Northern Territory, but again lacked intermediate sampling (Broderick et al., 2011).

The samples from of Wy at the mouth of the Cambridge Gulf within the broader Joseph Bonaparte Gulf (JBG) were distinct from CS in the Kimberley region. The hydrodynamics of the JBG, in particular the effect of the seasonal current flow and the high bottom stress, are likely to be factors linked to the isolation of Wy from the rest of the Kimberley area (Condie, 2011). The geographic distance between CS and Wy may be at least in part responsible for this genetic gap, recognizing that intermediate sampling between these locations may have identified additional population subdivision. Wy is part of a genetically homogenous group that spans the western side of the Northern Territory. An explanation for the isolation of the Northern Territory group is the year‐round persistent Arnhem Current (Condie, 2011) that effectively isolates waters across the Northern Territory.

The VI are situated at the western base of the Gulf of Carpentaria, another area dominated by specific circulation flows. The Gulf is a shallow semi‐enclosed sea with very long flushing times and long residence times for particles (Condie, 2011). The water circulation in the Gulf is triggered by a seasonal change in the wind direction. Transport in and out of the Gulf of Carpentaria is influenced by seasonal wind variations as well as the westward Arnhem Current that offsets transport into the Gulf during the summer monsoon season (Condie, 2011). Moreover, the southern part of the Gulf of Carpentaria is even more isolated from the Arafura and Timor seas than the northern section as each part of the Gulf exhibits its own seasonal current pattern. The current flow, through the transportation of presettlement life stages such as eggs and larvae, could account for the separation of the VI from the remaining Northern Territory locations. Conversely, in the study of *S. semifasciatus*, individuals from the VI were grouped with locations on the Northern Territory coast and not with the other eastern Gulf of Carpentaria locations in the Queensland jurisdiction (Broderick et al., 2011).

The offshore population of the AS off the coast of Arnhem Land and close to the Wessel Islands was a genetically isolated population. The distance from the shore and the greater depths may prevent this population from exchanging individuals with coastal locations, which would explain the distinctiveness of that population. However, there is a significant information gap about this population and its degree of dependence upon other inshore populations of Australia or Papua, which requires further investigation.

### 4.3 Integration across methods: example of RB and CS locations

Despite the overall evidence of a strong genetic break, Bayesian analysis at the individual level showed that RB consisted of an admixed population, with some fish having genotypes similar to CS individuals (Table 5). Ocean currents can greatly influence recruitment patterns of organisms through the dispersal of propagules (e.g., Feutry et al., 2013). Modelling of water currents along the northern Australian coast has shown that the “Indonesian Through Flow” influences the major seasonal southward surface current along the northern Western Australia coast (known as the “Holloway Current”) (D'Adamo, Fandry, Buchan, & Domingues, 2009). This narrow boundary current, that mainly influences the continental shelf in the Canning and Kimberley bioregions and predominantly flows southwest throughout the year, albeit with seasonal variations (Bahmanpour, Pattiaratchi, Wijeratne, Steinberg, & D'Adamo, 2016), likely favours the southward dispersal of larvae from CS to RB (Travers et al., 2010). However, seasonal variation is also observed, with a northward flow of current during summer (January‐March) that contributes to water build‐up in the Gulf of Carpentaria. During the summer spawning months, *P. diacanthus* larvae from CS would mainly drift northward but could still drift southward to RB. The seasonality and direction of the Holloway current flow may explain the genetic heterogeneity of RB samples. Although most of the individuals from RB are close to maturity and possibly the oldest fish among our samples (mean total length 1,018 mm), Wy also has larger fish (mean total length 1,061 mm) and do not present such a genetic distinctiveness. The segregation of genotypes in the RB population did not seem to depend on the year of sampling nor to be linked to the fish age or cohort, although the limited temporal window of our sampling regime did not allow statistical testing of this hypothesis.

Using all three techniques to detect migrants through mis‐assignments tests for contemporary connectivity between locations. Neither the otolith or parasite data showed evidence of matching mis‐assignment to genotypes at the individual level between RB and CS locations. If the migrants arrived as juveniles, they would have the genetic signature of the source population, but as they mature in the new location, they would acquire the local parasite and otolith signature. If the migrants arrived as adults, they would have the genetic and near core signatures of the source population, and possibly intermediate parasite fauna composition. Alternatively, the genetically distinct immigrants could be the descendants of true immigrants—hence, they would be genetically distinct, but have the local otolith (near core and margin) and parasite signatures. The absence of a matching pattern between genetics and ecological methods in individual mis‐assignment reveals that the genetically mis‐assigned individuals from RB may (i) not be contemporary immigrants but rather a genetic signal of ancestral immigrants or (ii) be contemporary immigrants from sites not sampled in the study. Fine‐scale sampling and temporal series collection would be required to further investigate the processes that drive the genetic composition of fish in the RB population.

### 4.4 Management implications

The highly complex population structure of *P. diacanthus* shown by this study across northern Australia emphasizes the likely vulnerability of the species to overfishing, and importantly, guides the appropriate spatial scale for stock assessment and fisheries management. In particular, the population genetics and otolith and parasites data suggest that stock assessments and fisheries management on a spatial scale of as little as 100 s of km may be justified. Given genetic breaks among broad regions, it is unlikely that immigration of recruits from other regions would contribute to population recovery in the short term should one or more regions suffer severe population declines. At a finer scale, the parasite and otolith chemistry analyses suggest that movement of recruits among sites (e.g., reef complexes) is generally limited and that heavily depleted sites may be highly reliant on self‐recruitment for population maintenance and, therefore, may be slow to recover once depleted. Within the Western Australia fisheries management jurisdiction, RB, CS and Wy were genetically distinct from each other, indicating that location‐specific stock assessments and location‐specific management arrangements for *P. diacanthus* need to be considered by fisheries managers, or alternatively that the location‐specific “stocks” identified are considered within assessment, monitoring and management frameworks.

*Protonibea diacanthus* is targeted by commercial, recreational and indigenous fisheries, which makes population modelling challenging over limited spatial scales of 100 s of kms. The difficulty with modelling over this spatial scale is the logistical challenges associated with collecting matching stock‐specific data such as reproduction and spawning dynamics, growth rates, exploitation rates and natural mortality. This logistical challenge comes with a significant cost burden. These factors indicate that fine‐scale management is problematical once the additional cost of compliance, regulation and enforcement is added. Within that framework, consideration needs to be given to a range of other options that limit fishing effort, including limited entry, limiting fishing effort, consideration of targeted fisheries spatial closures, rotational harvests or a combination of these approaches with other traditional fisheries management arrangements (e.g., co‐operative management of local fisheries with Indigenous people). This approach has been used in the Northern Territory where a series of reef‐scale closure areas have been implemented which has subsequently shown to aid the recovery of *P. diacanthus* and *Lutjanus johnii* (NTG 2016). Consideration is also being given to developing fine‐scale areal catch limits for *P. diacanthus* based on information from this study (Thor Saunders pers. com) demonstrating that management at the scale of populations is achievable.

## 5 CONCLUSIONS

Several lines of evidence support a model of low dispersal in *P. diacanthus*. Significant spatial genetic population structure was supported by finer spatial‐scale parasite assemblage and otolith chemistry data. There is evidence of a small amount of migration—most likely in the pelagic larval phase—from adjacent populations corresponding to prevailing currents, which was inferred from integration across data from three sources.

Our finding that *P. diacanthus* is highly structured across northern Australia from RB to the VI has demonstrated that the combination of ecological and genetic markers provides a more holistic view of population structure when deployed together on the same set of individuals. Genetics provides the overall framework for applying ecological methods and provides also considerable information about exchange between populations. Sensitive ecological methods, such as otolith chemistry and parasites, provide resolution of the fine‐scale spatial separation within and between collection locations, but are unable to provide broader scale information over intergenerational timescales. Our results collectively demonstrate location‐specific fidelity in the juvenile and adult phases and suggest that dispersal is likely to occur primarily during the very early life history as eggs and/or pelagic larvae. Further research (e.g., acoustic telemetry) would be required to directly quantify the extent of site attachment across the life history of *P. diacanthus*.

The finding that *P. diacanthus* across north‐western Australia consists of a set of spatially structured populations with low connectivity sets a framework to develop approaches for the assessment and spatial management of the species. The collapse of the North Queensland stock (Phelan, Gribble, & Garrett, 2008) and stocks in India and China (James, 1994; Sadovy & Cheung, 2003) demonstrates that *P. diacanthus* is prone to local depletion and our findings suggest that this vulnerability is likely due to its low dispersal and high location‐specific fidelity. Stock assessment models for this species cannot assume free and frequent movement of individuals among the genetic populations stock boundaries. Because population recovery will mainly depend on limited interchange of early life‐stages such as eggs or larvae between neighbouring populations (stocks), it may take multiple generations to replenish depleted stocks and there is potential that some heavily depleted stocks may not recover at all.

## DATA ARCHIVING STATEMENT

Data files with individual information (genotypes, parasites assemblage and abundance and otoliths microchemistry) are available from DRYAD (https://doi.org/10.5061/dryad.9rs5d).

## ETHICS APPROVAL

This work was conducted under Charles Darwin University Animal Ethics permit A13014. Voucher specimens of parasites will be deposited within the collections of the Museum and Art Gallery of the Northern Territory (MAGNT), the Australian Helminthological Collection (AHC) and Marine Invertebrates section of the South Australian Museum (SAM), the Parasitology Collection of the Queensland Museum (QM), and the Natural History Museum of London (BMNH).
