# Supplementary material for: Strong population structure deduced from genetics, otolith chemistry and parasite abundances explains vulnerability to localized fishery collapse in a large Sciaenid fish, Protonibea diacanthus
Source: Evol Appl. 2017 Jul 12;10(10):978–93. doi: 10.1111/eva.12499 (PMC5680636; doi:10.1111/eva.12499)
Supplement: Supplementary file 1 [file EVA-10-978-s001.docx]

**Appendices for Manuscript entitled ‘*Strong population structure deduced from genetics, otolith chemistry and parasite abundances explains vulnerability to localised fishery collapse in a large Sciaenid fish, Protonibea diacanthus*’**

**TABLE OF CONTENT**

**Appendix S1: Methods 2**

**Appendix A1: Genetics methodology 2**

***STRUCTURE 2***

***DAPC 2***

**Appendix A2: Otolith chemistry methodology 3**

***Preparation and laser ablation 3***

***Data transformation 3***

**Appendix A3: Parasites methodology 4**

***Extraction of parasites 4***

**References 5**

**Appendix S2: Results 6**

**Appendix B1: Mean elemental ratio in the otolith 6**

**Appendix B2: Mean abundance and prevalence of parasites 7**

**Appendix B3: Summary statistics of microsatellite data 9**

**Appendix B4: Genetic Bayesian-based clustering analysis 13**

**Appendix B5: Individual assignments for otoliths and parasites 14**

**Appendix A1: Genetics methodology**

***STRUCTURE***

In STRUCTURE varying numbers of possible genetic clusters within the dataset were trialed from two to the number of sampling locations in the dataset (all locations dataset, K=2-11). The analysis was run 10 times for each K tested, with 100,000 runs for burn-in, followed by 500,000 replicates. Because admixture in marine population is a common characteristic (Porras-Hurtado et al., 2013), we performed the analysis using the *admixture* model and correlated allele frequencies (Falush et al., 2003). The model was run with and without the sampling location information (*LOCPRIOR* parameter)*.* This option is particularly informative when population structure signals are weak as it improves the program performance and ability to find the clusters (Hubisz et al., 2009). The results were visualized in Pophelper (Francis, 2016) and the optimal number of genetic clusters was estimated using the rate of change in the log probability of data between successive K values (∆K) as described by Evanno et al. (2005). However, given the large degree of uncertainty around the statistical estimation of K (Meirmans, 2015) we examined all the clustering patterns that warranted a biological interpretation.

***DAPC***

Evidence of genetic clusters was also examined in DAPC by running successive K-means clustering in the *find.clusters* function. We tested the same values of K as for STRUCTURE (*i.e.* K =2–11), with ten runs at each value of K. The optimal number of clusters was determined using the Bayesian Information Criterion (BIC) (*i.e.* K with the lowest BIC value is ideally the optimal number of clusters). However, BIC values may continue to decrease after the true K value in the case of genetic clines and hierarchical structure (Jombart et al., 2010). Therefore, the rate of decrease in BIC values was visually examined to identify values of K, after which BIC values decreased only subtly (Jombart et al., 2010). Once the number of genetic clusters was selected, the dataset was sub-divided into locations or groups of spatially continuous locations (the number of groups equal to the number of genetic clusters identified) by taking into account the broad-scale STRUCTURE results. The DAPC function was then executed using this grouping. The optimal number of Principal Components (PCs) to retain was assessed with a cross-validation approach in *adegenet* using the *xvalDapc* function. The result of the DAPC was presented in an ordination plot with the first two axes.

**Appendix A2: Otolith chemistry methodology**

***Preparation and laser ablation***

The left sagittal otolith was selected from each individual and embedded in epoxy resin and then sectioned to 350 µm thickness using a low speed saw. Sections were polished with diamond lapping film (30, 9 and 3 µm), mounted on microscope slides using epoxy resin, triple-rinsed with Milli-Q water and then allowed to dry in a laminar flow cabinet. Elemental analysis was performed using an Agilent 7700x quadrupole inductively coupled plasma mass spectrometer (ICP-MS) coupled to a custom built RESOlution laser ablation system with a HelEx cell and Compex 110 ArF excimer laser. The laser was operated using a spot size of 72 μm diameter with laser energy at 2.7 J/cm^2^ and a repetition rate of 5 Hz. Ablations occurred inside a sealed chamber in an atmosphere of pure He with the ablated material being transported to the ICPMS in the Ar carrier gas. The laser ablation spot sample consisted of a 20 second blank, followed by an ablation period of 50 seconds, of which the first 5 seconds and the last second were excluded from data integration to allow for signal stabilisation.

***Data transformation***

Data reduction and processing was completed using IOLITE version 3 (Paton et al., 2011). Subtraction of background ion counts from otolith counts was followed by the normalization of each element to ^43^Ca; and the National Institute of Standards and Technology glass standard (NIST 612) was used as the external calibration standard which was analysed after every 10 otolith samples to correct for any long-term drift in the instrument. The limits of detection (LOD) were calculated for each sample from the ablation yield equivalent to 3x standard deviation (SD) of the blank background measurements. Concentrations of ^23^Al, ^49^Ti and ^53^Cr were mostly <LOD and were not included in the analysis. Detection limits and percentage of otolith ablations >LOD as well as estimated instrument precision (% Relative Standard Deviation [RSD] based on analyses of NIST 612 and MACS-3 reference standards) for each trace element analysed at all two ablation zones are presented below. For all elements, the ratio of element isotope intensity to Ca intensity was used to estimate the element:^43^Ca ratio. These ratios were converted to molar ratios and were expressed as element:Ca molar ratios in mmol.mol^-1^ or µmol.mol^-1^.

**Appendix A3: Parasites methodology**

***Extraction of parasites***

After defrosting, the gills were separated into individual arches, washed in water, and examined. The stomach and intestinal tract were separated from the mesenteries and associated organs, slit along their length and washed for parasite examination. The supernatant of the washings were decanted and the sediment searched for parasites. The separated mesenteries were washed and examined; encysted parasites were removed from the mesenteries and released from their associated cysts prior to fixation. For female fish, ovaries were slit along their length and examined under a dissecting microscope for the presence of philometrid nematodes. Parasites were identified to the lowest taxonomic resolution possible.

**References**

Evanno, G., Regnaut, S., & Goudet, J. (2005). Detecting the number of clusters of individuals using the software STRUCTURE: a simulation study. *Molecular ecology, 14*, 2611-2620.

Falush, D., Stephens, M., & Pritchard, J. K. (2003). Inference of population structure using multilocus genotype data: linked loci and correlated allele frequencies. *Genetics, 164*, 1567-1587.

Francis, R. M. (2016). POPHELPER: an R package and web app to analyse and visualize population structure. *Molecular ecology resources*. doi: 10.1111/1755-0998.12509

Hubisz, M. J., Falush, D., Stephens, M., & Pritchard, J. K. (2009). Inferring weak population structure with the assistance of sample group information. *Molecular ecology resources, 9*, 1322-1332.

Jombart, T., Devillard, S., & Balloux, F. (2010). Discriminant analysis of principal components: a new method for the analysis of genetically structured populations. *BMC genetics, 1*, 1-15.

Paton, C., Hellstrom, J., Paul, B., Woodhead, J., & Hergt, J. (2011). Iolite: Freeware for the visualisation and processing of mass spectrometric data. *Journal of Analytical Atomic Spectrometry, 26*, 2508-2518.

Porras-Hurtado, L., Ruiz, Y., Santos, C., Phillips, C., Carracedo, Á., & Lareu, M. (2013). An overview of STRUCTURE: applications, parameter settings, and supporting software. *Frontiers in Genetics*, *4*, 98.

**Appendix B1: Mean elemental ratio in the otolith**

Mean concentrations (± s.e.) of element:Ca ratios from the near core (light grey bars) and margin (dark grey bars) of *Protonibea diacanthus* otoliths collected from 11 locations across northern Australia. Location codes follow Table 1.


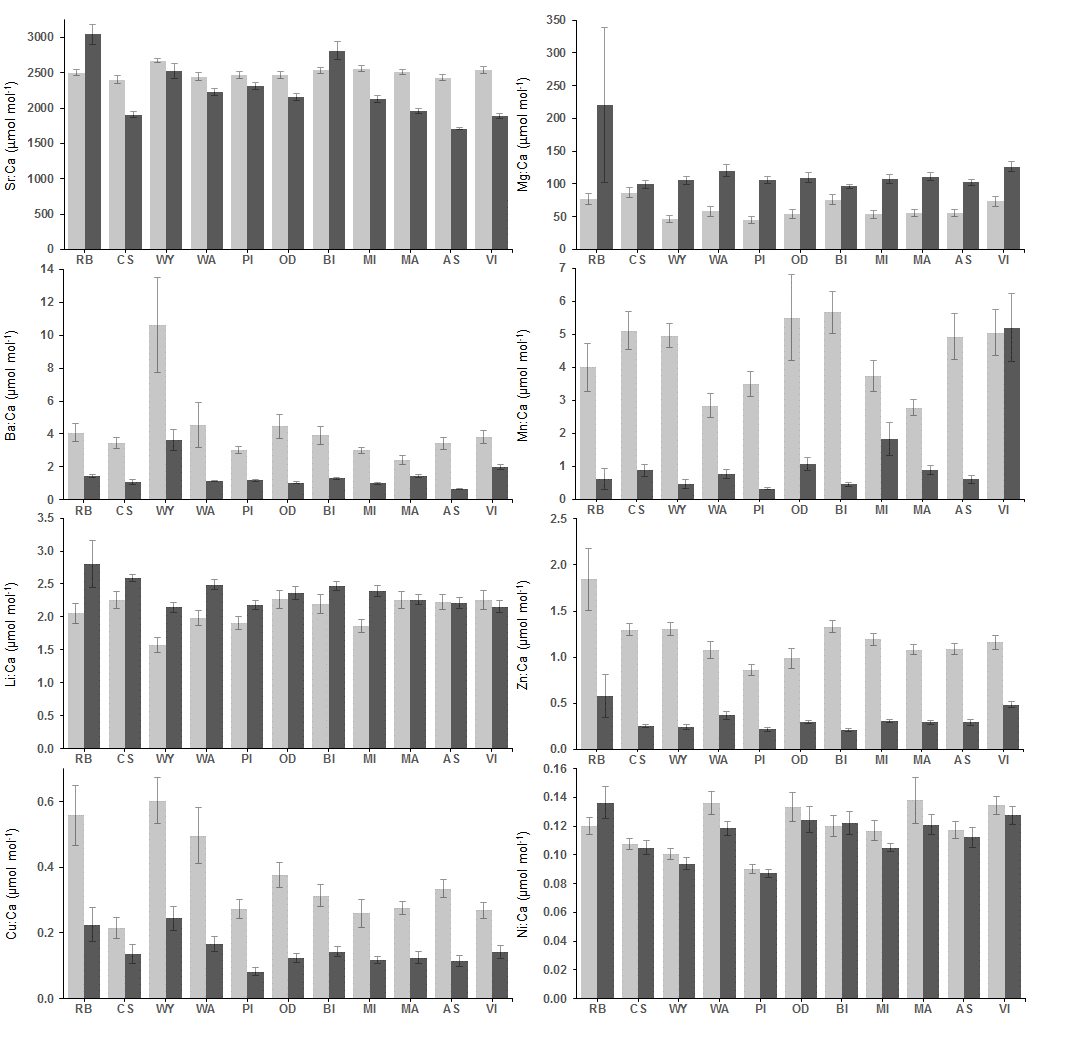


**Appendix B2: Mean abundance and prevalence of parasites**

Parasites found infecting *Protonibea diacanthus* sampled from 11 locations across northern Australia used in the analyses for this study. Data is presented as mean abundance with prevalence in parentheses. Data presented is untransformed. Only parasites used in analyses are included.

|  | **Collection Locations** | **RB** | **CS** | **Wy** | **Wa** | **PI** | **OD** | **BI** | **MI** | **Ma** | **AS** | **VI** |
| --- | --- | --- | --- | --- | --- | --- | --- | --- | --- | --- | --- | --- |
|  | Sample Size | 36 | 20 | 34 | 25 | 22 | 17 | 28 | 30 | 29 | 19 | 29 |
| Gills | *Caligus haemulonis* & *Caligus* sp. 2 combined | 0.3 (19) |  | 0.2 (9) | 0.04 (4) | 0.5 (32) | 0.1 (6) | 0.04 (4) | 0.1 (10) | 3.2 (59) |  | 0.1 (7) |
|  | *Caligus* sp. 3 |  |  |  |  |  |  | 0.3 (18) |  |  |  |  |
|  | *Lernanthropus cruciatus* | 4.6 (83) |  | 0.9 (32) | 2.2 (56) | 1.4 (59) | 0.4 (24) | 6.3 (82) | 1.2 (43) | 1.4 (59) | 1.0 (42) | 2.3 (86) |
|  | Acanthocolpidae metacercaria | 0.5 (22) | 0.6 (10) |  |  |  |  |  | 0.7 (10) | 0.1 (14) |  | 3.3 (41) |
| Body cavity, mesenteries, tissues & ovaries | *Pseudogilquinia* sp. |  | 0.1 (10) | 0.3 (6) |  |  |  | 0.04 (4) | 0.03 (3) |  |  |  |
|  | *Pterobothrium* sp. 1 | 0.6 (22) | 1.6 (50) | 9.3 (62) | 3.0 (24) | 9.7 (96) | 1.1 (29) | 5.5 (79) | 1.3 (23) | 0.03 (3) | 0.3 (21) |  |
|  | *Pterobothrium* sp. 3 | 0.1 (11) | 0.3 (20) | 3.6 (62) | 0.2 (12) | 0.2 (9) |  | 0.3 (14) | 0.03 (3) | 0.03 (3) |  |  |
|  | *Pterobothrium* sp. 5 | 0.1 (6) | 0.1 (5) | 0.4 (18) | 0.2 (12) | 0.1 (5) |  | 0.2 (11) | 0.1 (7) | 0.1 (3) |  |  |
|  | *Otobothrium* sp. |  | 0.1 (5) | 0.1 (9) | 0.04 (4) | 0.2 (14) |  | 0.2 (21) | 0.1 (10) | 0.03 (3) |  |  |
|  | *Paratobothrium* sp. | 9 (64) |  | 0.7 (21) |  | 0.1 (5) |  |  |  | 0.5 (24) |  |  |
|  | *Nybelinia* spp. | 0.1 (8) | 0.1 (5) | 0.1 (9) | 0.04 (4) | 0.1 (5) | 1.5 (29) | 0.1 (7) | 0.2 (17) | 0.03 (3) | 0.1 (5) | 0.03 (3) |
|  | *Dasyrhynchus* sp. | 0.3 (19) |  | 19.9 (97) | 3.2 (76) | 3.0 (77) |  | 9.6 (75) | 1.6 (17) | 0.1 (3) |  |  |
|  | *Poecilanstrium* sp. | 7.9 (92) | 3.1 (80) | 5.7 (85) | 4.7 (92) | 12.1 (100) | 1.8 (47) | 11.4 (89) | 3.2 (50) | 0.6 (28) | 0.4 (32) | 0.2 (17) |
|  | *Callitetrarhynchus* sp. | 1.4 (61) | 0.1 (50) | 0.4 (27) | 0.7 (24) | 2.0 (64) | 0.1 (12) | 2.3 (68) | 0.6 (17) | 0.1 (14) | 10.4 (90) | 0.3 (24) |
|  | *Philometra protonibeae* | 0.04 (4.0) | 0.1 (12.5) | 0.18 (23.1) | 0.12 (25.0) | 0.41 (69.2) |  | 0.36 (66.7) | 0.13 (33.3) | 0.03 (8.3) |  |  |
|  | *Philometroides* n. sp. 2 |  |  |  |  |  |  |  | 0.2 (3) |  | 0.3 (32) |  |
|  | Gnathostomidae sp. | 1.4 (53) |  | 1.1 (35) |  | 0.4 (18) |  | 0.4 (14) | 0.1 (3) | 0.6 (38) |  |  |
|  | *Serrasentis sagittifer* | 0.1 (3) |  |  | 0.2 (8) | 0.1 (9) |  |  | 0.5 (33) | 0.2 (21) | 0.8 (53) | 0.2 (17) |
|  | Sebekidae spp. |  |  | 0.8 (24) |  |  |  | 0.1 (4) |  | 0.03 (3) |  |  |
|  | Pseudoparasitic eel (Fam. Opichthidae) | 0.6 (36) | 0.1 (10) | 0.1 (6) |  |  |  |  |  | 0.03 (3) |  |  |
| Intestinal canal | *Orientodiploproctodaeum* sp. | 12.2 (86) | 7.8 (95) | 24.7 (88) | 46.8 (76) | 54.4 (96) | 8.8 (71) | 52.7 (93) | 28.8 (67) | 6.8 (90) | 0.1 (5) | 22.0 (100) |
|  | *Pleorchis* sp. | 1.3 (36) | 0.1 (5) |  | 0.5 (20) |  | 2.9 (47) | 0.2 (7) | 1.8 (47) | 0.1 (7) | 0.3 (11) |  |
|  | *Stephanostomum* sp. | 0.5 (11) | 2 (65) | 3.2 (38) | 10.2 (92) | 6.5 (59) | 10.4 (35) | 21.5 (75) | 5.0 (67) | 0.1 (10) | 0.1 (5) | 1.8 (41) |
|  | Hemiuridae sp. | 7.4 (64) | 0.1 (5) |  |  | 0.1 (5) | 0.1 (6) | 0.1 (4) | 0.3 (23) | 0.03 (3) |  | 0.3 (21) |
|  | *Dichelyne spinigerus* |  | 1.3 (65) | 6.7 (62) | 13.2 (88) | 4.1 (73) | 10.0 (71) | 9.0 (79) | 4.5 (73) |  |  | 0.1 (10) |
|  | Camallanidae sp. |  |  | 0.1 (12) | 0.04 (4) | 0.1 (5) |  |  | 0.1 (10) |  |  |  |
|  | Ascaridae sp. 1 |  |  | 0.41 (20.6) |  |  |  |  |  |  |  |  |
|  | Ascaridae sp. 2 |  |  |  | 0.2 (8) | 0.1 (9) |  | 0.04 (4) | 0.4 (23) | 12.2 (72) | 5.5 (11) | 0.03 (3) |
|  | Acanthocephala | 0.1 (6) |  | 0.4 (12) |  |  | 0.1 (6) |  |  | 0.03 (3) | 0.1 (5) |  |

**Appendix B3: Summary statistics of microsatellite data**

Summary statistics of 11 microsatellite loci for *Protonibeas diacanthus* from 284 individuals sampled from 11 locations across northern Australia. *n* is the sample size, *#A* is the number of alleles, *A_R_* is the allelic richness, *I* is the Shannon’s information index, *H_E_* is the expected heterozygosity, *H_O_* is the observed heterozygosity, *F* is the fixation index (*H_E_*-*H_O_*)/*H_E_*, and p-value of the Hardy-Weinberg exact test.

| **Pop** |  | ***Prd044*** | ***Prd023*** | ***Prd042*** | ***Prd012*** | ***Prd046*** | ***Prd018*** | ***Prd020*** | ***Prd045*** | ***Prd049*** | ***Prd036*** | ***Prd024*** |
| --- | --- | --- | --- | --- | --- | --- | --- | --- | --- | --- | --- | --- |
| **RB** | ***n*** | 32 | 32 | 32 | 29 | 32 | 30 | 32 | 32 | 32 | 32 | 32 |
|  | ***#A*** | 5 | 8 | 5 | 12 | 4 | 12 | 6 | 6 | 3 | 6 | 5 |
|  | ***A_R_*** | 3.298 | 5.044 | 4.154 | 6.648 | 1.645 | 6.081 | 3.543 | 1.533 | 1.171 | 1.653 | 3.225 |
|  | ***I*** | 1.344 | 1.805 | 1.487 | 2.124 | 0.777 | 2.057 | 1.418 | 0.744 | 0.313 | 0.854 | 1.286 |
|  | ***H_O_*** | 0.594 | 0.719 | 0.625 | 0.759 | 0.375 | 0.833 | 0.781 | 0.344 | 0.094 | 0.281 | 0.656 |
|  | ***H_E_*** | 0.697 | 0.802 | 0.759 | 0.850 | 0.392 | 0.836 | 0.718 | 0.348 | 0.146 | 0.395 | 0.690 |
|  | ***F*** | 0.148 | 0.104 | 0.177 | 0.107 | 0.044 | 0.003 | -0.088 | 0.011 | 0.358 | 0.288 | 0.049 |
|  | ***p-value*** | 0.001 | 0.044 | 0.211 | 0.093 | 0.157 | 0.183 | 0.679 | 0.161 | 0.155 | 0.006 | 0.182 |
| **CS** | ***n*** | 18 | 18 | 18 | 15 | 18 | 14 | 18 | 13 | 18 | 18 | 18 |
|  | ***#A*** | 8 | 7 | 6 | 10 | 4 | 8 | 7 | 6 | 4 | 6 | 5 |
|  | ***A_R_*** | 4.025 | 5.684 | 3.340 | 6.164 | 1.333 | 5.600 | 2.746 | 2.641 | 1.493 | 2.365 | 3.812 |
|  | ***I*** | 1.630 | 1.850 | 1.409 | 2.018 | 0.535 | 1.871 | 1.348 | 1.302 | 0.647 | 1.091 | 1.421 |
|  | ***H_O_*** | 0.778 | 0.944 | 0.667 | 0.933 | 0.278 | 0.571 | 0.667 | 0.769 | 0.278 | 0.444 | 0.722 |
|  | ***H_E_*** | 0.752 | 0.824 | 0.701 | 0.838 | 0.250 | 0.821 | 0.636 | 0.621 | 0.330 | 0.577 | 0.738 |
|  | ***F*** | -0.035 | -0.146 | 0.048 | -0.114 | -0.111 | 0.304 | -0.049 | -0.238 | 0.159 | 0.230 | 0.021 |
|  | ***p-value*** | 0.649 | 0.671 | 0.747 | 0.803 | 1.000 | 0.017 | 0.771 | 0.928 | 0.512 | 0.066 | 0.902 |
| **Wy** | ***n*** | 34 | 34 | 34 | 32 | 34 | 34 | 33 | 33 | 34 | 34 | 34 |
|  | ***#A*** | 8 | 8 | 5 | 16 | 5 | 8 | 8 | 5 | 4 | 10 | 6 |
|  | ***A_R_*** | 3.932 | 5.598 | 3.095 | 9.846 | 1.609 | 4.797 | 4.445 | 2.344 | 1.127 | 3.163 | 3.238 |
|  | ***I*** | 1.564 | 1.854 | 1.286 | 2.488 | 0.817 | 1.779 | 1.714 | 1.109 | 0.285 | 1.517 | 1.417 |
|  | ***H_O_*** | 0.735 | 0.794 | 0.824 | 0.906 | 0.324 | 0.971 | 0.970 | 0.515 | 0.118 | 0.765 | 0.618 |
|  | ***H_E_*** | 0.746 | 0.821 | 0.677 | 0.898 | 0.378 | 0.792 | 0.775 | 0.573 | 0.113 | 0.684 | 0.691 |
|  | ***F*** | 0.014 | 0.033 | -0.217 | -0.009 | 0.145 | -0.226 | -0.251 | 0.102 | -0.042 | -0.118 | 0.106 |
|  | ***p-value*** | 0.459 | 0.897 | 0.479 | 0.779 | 0.079 | 0.743 | 0.157 | 0.055 | 1.000 | 0.774 | 0.056 |
| **Wa** | ***n*** | 25 | 25 | 25 | 23 | 25 | 25 | 25 | 23 | 25 | 25 | 25 |
|  | ***#A*** | 8 | 8 | 5 | 12 | 4 | 7 | 7 | 7 | 4 | 7 | 5 |
|  | ***A_R_*** | 4.340 | 5.580 | 3.247 | 8.397 | 1.649 | 3.788 | 3.541 | 2.388 | 1.337 | 2.706 | 2.577 |
|  | ***I*** | 1.690 | 1.871 | 1.348 | 2.263 | 0.759 | 1.511 | 1.477 | 1.208 | 0.539 | 1.331 | 1.178 |
|  | ***H_O_*** | 0.720 | 0.840 | 0.680 | 0.739 | 0.440 | 0.680 | 0.720 | 0.609 | 0.280 | 0.640 | 0.720 |
|  | ***H_E_*** | 0.770 | 0.821 | 0.692 | 0.881 | 0.394 | 0.736 | 0.718 | 0.581 | 0.252 | 0.630 | 0.612 |
|  | ***F*** | 0.064 | -0.023 | 0.017 | 0.161 | -0.118 | 0.076 | -0.003 | -0.047 | -0.111 | -0.015 | -0.176 |
|  | ***p-value*** | 0.829 | 0.018 | 0.258 | 0.019 | 0.649 | 0.129 | 0.615 | 0.402 | 1.000 | 0.565 | 0.726 |
| **PI** | ***n*** | 29 | 28 | 29 | 25 | 29 | 28 | 29 | 26 | 29 | 29 | 29 |
|  | ***#A*** | 8 | 8 | 5 | 15 | 5 | 7 | 8 | 5 | 4 | 7 | 6 |
|  | ***A_R_*** | 4.083 | 5.560 | 3.260 | 8.993 | 2.541 | 4.994 | 3.995 | 1.791 | 1.330 | 2.817 | 2.288 |
|  | ***I*** | 1.649 | 1.873 | 1.352 | 2.422 | 1.220 | 1.717 | 1.571 | 0.905 | 0.525 | 1.312 | 1.101 |
|  | ***H_O_*** | 0.759 | 0.786 | 0.690 | 1.000 | 0.690 | 0.857 | 0.862 | 0.538 | 0.276 | 0.724 | 0.552 |
|  | ***H_E_*** | 0.755 | 0.820 | 0.693 | 0.889 | 0.606 | 0.800 | 0.750 | 0.442 | 0.248 | 0.645 | 0.563 |
|  | ***F*** | -0.005 | 0.042 | 0.005 | -0.125 | -0.137 | -0.072 | -0.150 | -0.219 | -0.113 | -0.123 | 0.020 |
|  | ***p-value*** | 0.897 | 0.706 | 0.052 | 0.860 | 0.883 | 0.910 | 0.040 | 0.895 | 1.000 | 0.901 | 0.021 |
| **OD** | ***n*** | 16 | 16 | 16 | 14 | 16 | 16 | 16 | 13 | 16 | 16 | 16 |
|  | ***#A*** | 6 | 8 | 4 | 12 | 4 | 7 | 8 | 3 | 4 | 7 | 5 |
|  | ***A_R_*** | 4.031 | 5.953 | 2.926 | 9.116 | 1.690 | 4.697 | 5.224 | 1.888 | 1.213 | 2.829 | 2.522 |
|  | ***I*** | 1.559 | 1.906 | 1.156 | 2.333 | 0.787 | 1.701 | 1.828 | 0.821 | 0.414 | 1.434 | 1.147 |
|  | ***H_O_*** | 0.813 | 0.875 | 0.438 | 1.000 | 0.500 | 0.750 | 0.563 | 0.462 | 0.188 | 0.563 | 0.688 |
|  | ***H_E_*** | 0.752 | 0.832 | 0.658 | 0.890 | 0.408 | 0.787 | 0.809 | 0.470 | 0.176 | 0.646 | 0.604 |
|  | ***F*** | -0.081 | -0.052 | 0.335 | -0.123 | -0.225 | 0.047 | 0.304 | 0.019 | -0.067 | 0.130 | -0.139 |
|  | ***p-value*** | 0.915 | 0.818 | 0.018 | 0.929 | 1.000 | 0.771 | 0.026 | 0.526 | 1.000 | 0.062 | 0.478 |
| **BI** | ***n*** | 28 | 28 | 28 | 24 | 28 | 28 | 28 | 28 | 28 | 28 | 28 |
|  | ***#A*** | 9 | 8 | 5 | 15 | 3 | 7 | 9 | 4 | 4 | 6 | 6 |
|  | ***A_R_*** | 4.272 | 6.938 | 3.621 | 9.600 | 1.602 | 3.778 | 4.000 | 1.570 | 1.344 | 2.708 | 3.200 |
|  | ***I*** | 1.749 | 1.997 | 1.383 | 2.441 | 0.667 | 1.566 | 1.682 | 0.725 | 0.539 | 1.271 | 1.377 |
|  | ***H_O_*** | 0.750 | 0.821 | 0.679 | 0.917 | 0.393 | 0.679 | 0.714 | 0.357 | 0.286 | 0.750 | 0.643 |
|  | ***H_E_*** | 0.766 | 0.856 | 0.724 | 0.896 | 0.376 | 0.735 | 0.750 | 0.363 | 0.256 | 0.631 | 0.688 |
|  | ***F*** | 0.021 | 0.040 | 0.063 | -0.023 | -0.046 | 0.077 | 0.048 | 0.016 | -0.117 | -0.189 | 0.065 |
|  | ***p-value*** | 0.485 | 0.015 | 0.751 | 0.665 | 1.000 | 0.303 | 0.712 | 0.777 | 1.000 | 0.321 | 0.749 |
| **MI** | ***n*** | 29 | 29 | 29 | 21 | 29 | 29 | 29 | 29 | 29 | 29 | 29 |
|  | ***#A*** | 8 | 9 | 5 | 10 | 4 | 7 | 7 | 5 | 4 | 6 | 6 |
|  | ***A_R_*** | 4.174 | 6.444 | 3.419 | 7.056 | 1.606 | 4.034 | 3.384 | 2.222 | 1.194 | 3.311 | 2.580 |
|  | ***I*** | 1.673 | 1.960 | 1.338 | 2.086 | 0.753 | 1.594 | 1.388 | 1.102 | 0.385 | 1.382 | 1.189 |
|  | ***H_O_*** | 0.724 | 0.931 | 0.793 | 0.857 | 0.414 | 0.759 | 0.828 | 0.655 | 0.138 | 0.966 | 0.690 |
|  | ***H_E_*** | 0.760 | 0.845 | 0.707 | 0.858 | 0.378 | 0.752 | 0.705 | 0.550 | 0.162 | 0.698 | 0.612 |
|  | ***F*** | 0.048 | -0.102 | -0.121 | 0.001 | -0.096 | -0.009 | -0.175 | -0.191 | 0.150 | -0.383 | -0.126 |
|  | ***p-value*** | 0.663 | 0.826 | 0.889 | 0.859 | 0.389 | 0.297 | 0.539 | 0.982 | 0.171 | 0.077 | 0.457 |
| **Ma** | ***n*** | 25 | 25 | 25 | 19 | 25 | 25 | 24 | 24 | 25 | 25 | 25 |
|  | ***#A*** | 10 | 8 | 5 | 13 | 5 | 8 | 6 | 5 | 4 | 6 | 6 |
|  | ***A_R_*** | 4.386 | 6.158 | 3.264 | 9.890 | 2.189 | 4.562 | 2.946 | 2.931 | 1.178 | 2.927 | 2.828 |
|  | ***I*** | 1.781 | 1.918 | 1.335 | 2.407 | 1.061 | 1.733 | 1.262 | 1.307 | 0.362 | 1.331 | 1.306 |
|  | ***H_O_*** | 0.720 | 0.880 | 0.720 | 0.684 | 0.680 | 0.760 | 0.583 | 0.625 | 0.160 | 0.680 | 0.560 |
|  | ***H_E_*** | 0.772 | 0.838 | 0.694 | 0.899 | 0.543 | 0.781 | 0.661 | 0.659 | 0.151 | 0.658 | 0.646 |
|  | ***F*** | 0.067 | -0.051 | -0.038 | 0.239 | -0.252 | 0.027 | 0.117 | 0.051 | -0.058 | -0.033 | 0.134 |
|  | ***p-value*** | 0.051 | 0.445 | 0.705 | 0.001 | 0.482 | 0.015 | 0.541 | 0.201 | 1.000 | 0.913 | 0.063 |
| **AS** | ***n*** | 19 | 19 | 19 | 17 | 19 | 19 | 19 | 19 | 19 | 19 | 19 |
|  | ***#A*** | 8 | 7 | 5 | 11 | 4 | 11 | 7 | 8 | 4 | 8 | 5 |
|  | ***A_R_*** | 5.554 | 5.597 | 3.703 | 7.811 | 1.462 | 5.388 | 4.102 | 4.376 | 1.660 | 3.267 | 3.861 |
|  | ***I*** | 1.859 | 1.820 | 1.414 | 2.209 | 0.624 | 1.966 | 1.620 | 1.700 | 0.799 | 1.538 | 1.431 |
|  | ***H_O_*** | 0.789 | 0.842 | 0.632 | 0.882 | 0.211 | 0.789 | 1.000 | 0.737 | 0.474 | 0.842 | 0.947 |
|  | ***H_E_*** | 0.820 | 0.821 | 0.730 | 0.872 | 0.316 | 0.814 | 0.756 | 0.771 | 0.398 | 0.694 | 0.741 |
|  | ***F*** | 0.037 | -0.025 | 0.135 | -0.012 | 0.333 | 0.031 | -0.322 | 0.045 | -0.192 | -0.214 | -0.279 |
|  | ***p-value*** | 0.516 | 0.906 | 0.273 | 0.841 | 0.096 | 0.592 | 0.043 | 0.850 | 1.000 | 0.249 | 0.291 |
| **VI** | ***n*** | 28 | 29 | 29 | 26 | 29 | 29 | 28 | 28 | 29 | 29 | 29 |
|  | ***#A*** | 11 | 8 | 5 | 16 | 4 | 6 | 6 | 7 | 3 | 8 | 6 |
|  | ***A_R_*** | 5.917 | 5.881 | 2.905 | 7.308 | 1.528 | 4.092 | 3.240 | 3.039 | 1.503 | 3.780 | 2.813 |
|  | ***I*** | 2.005 | 1.877 | 1.208 | 2.341 | 0.659 | 1.533 | 1.320 | 1.425 | 0.569 | 1.634 | 1.220 |
|  | ***H_O_*** | 0.750 | 0.862 | 0.724 | 0.846 | 0.310 | 0.690 | 0.821 | 0.679 | 0.276 | 0.793 | 0.759 |
|  | ***H_E_*** | 0.831 | 0.830 | 0.656 | 0.863 | 0.345 | 0.756 | 0.691 | 0.671 | 0.335 | 0.735 | 0.644 |
|  | ***F*** | 0.097 | -0.039 | -0.104 | 0.020 | 0.102 | 0.087 | -0.188 | -0.011 | 0.176 | -0.078 | -0.177 |
|  | ***p-value*** | 0.175 | 0.601 | 0.800 | 0.140 | 0.404 | 0.158 | 0.237 | 0.275 | 0.398 | 0.741 | 0.649 |
| **All populations** | ***n*** | 283 | 283 | 284 | 245 | 284 | 277 | 281 | 268 | 284 | 284 | 284 |
|  | ***#A*** | 14 | 9 | 7 | 21 | 5 | 14 | 12 | 10 | 6 | 10 | 6 |
|  | ***A_R_*** | 4.365 | 5.858 | 3.358 | 8.257 | 1.714 | 4.710 | 3.742 | 2.429 | 1.323 | 2.866 | 2.995 |
|  | ***I*** | 1.682 | 1.885 | 1.338 | 2.285 | 0.787 | 1.730 | 1.512 | 1.123 | 0.489 | 1.336 | 1.279 |
|  | ***H_O_*** | 0.739 | 0.845 | 0.679 | 0.866 | 0.419 | 0.758 | 0.774 | 0.572 | 0.233 | 0.677 | 0.687 |
|  | ***H_E_*** | 0.765 | 0.828 | 0.699 | 0.876 | 0.399 | 0.783 | 0.724 | 0.550 | 0.233 | 0.636 | 0.657 |
|  | ***F*** | 0.034 | -0.020 | 0.027 | 0.011 | -0.033 | 0.031 | -0.069 | -0.042 | 0.013 | -0.046 | -0.046 |

**Appendix B4: Genetic Bayesian-based clustering analysis**

Results from the Bayesian population assignment of microsatellite data from *Protonibea diacanthus* using the software STRUCTURE a) without LOCPRIOR b) with LOCPRIOR. Location prefixes follow Table 1. Each vertical line represents an individual and the posterior probability proportions of its genotype assigned to the different genetic clusters. The number of genetic clusters tested ranges from K=2 to K=11; each plot represents one tested K. Population information was used as a prior in the analysis.

**
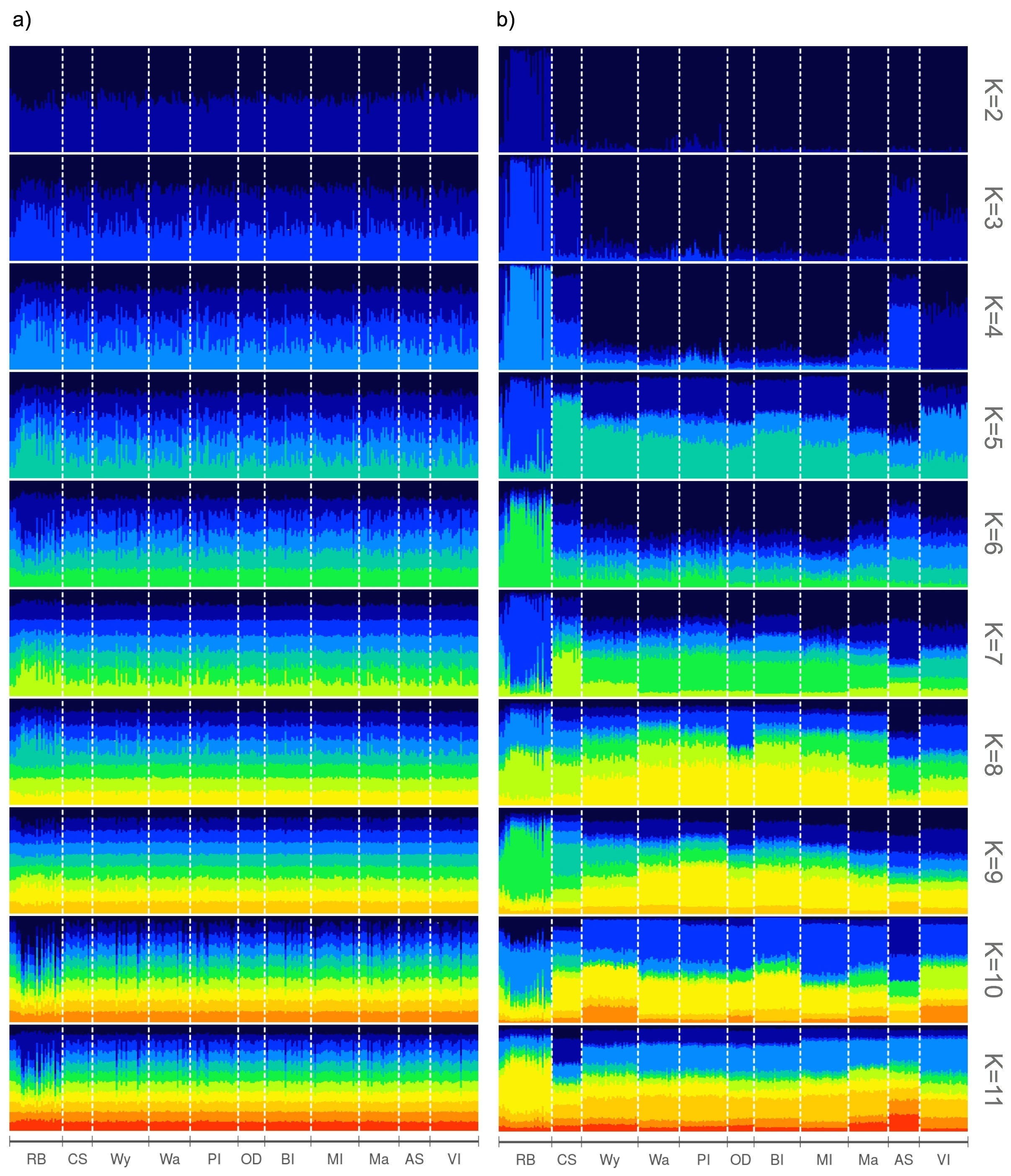
**

**Appendix B5: Individual assignments for otoliths and parasites**

Bayesian individual assignments within a) the Western region, b) the Darwin region and c) the Arnhem/Gulf region

**a)**

|  |  |  |  | **Assigned location** | | | | | | | | | | | | | |
| --- | --- | --- | --- | --- | --- | --- | --- | --- | --- | --- | --- | --- | --- | --- | --- | --- | --- |
|  |  |  |  | **Otolith Near Core** | | | |  | **Otolith Margin** | | | |  | **Parasites** | | | |
| **Sample** | **Size (mm)** | **Sampling location** |  | **CS** | **RB** | **Wa** | **Wy** |  | **CS** | **RB** | **Wa** | **Wy** |  | **CS** | **RB** | **Wa** | **Wy** |
| 2523 | 520 | CS |  | ✔ |  |  |  |  |  |  |  | ✔ |  | ✔ |  |  |  |
| 2510 | 524 | CS |  | ✔ |  |  |  |  | ✔ |  |  |  |  | ✔ |  |  |  |
| 2511 | 539 | CS |  | ✔ |  |  |  |  |  |  | ✔ |  |  | ✔ |  |  |  |
| 2516 | 565 | CS |  | ✔ |  |  |  |  | ✔ |  |  |  |  | ✔ |  |  |  |
| 2505 | 580 | CS |  | ✔ |  |  |  |  |  |  | ✔ |  |  | ✔ |  |  |  |
| 2508 | 594 | CS |  | ✔ |  |  |  |  |  |  |  | ✔ |  | ✔ |  |  |  |
| 2514 | 595 | CS |  | ✔ |  |  |  |  |  |  | ✔ |  |  |  |  | ✔ |  |
| 2515 | 595 | CS |  | ✔ |  |  |  |  | ✔ |  |  |  |  | ✔ |  |  |  |
| 2520 | 601 | CS |  | NA | NA | NA | NA |  | NA | NA | NA | NA |  | ✔ |  |  |  |
| 2504 | 623 | CS |  |  |  | ✔ |  |  | ✔ |  |  |  |  | ✔ |  |  |  |
| 2517 | 631 | CS |  |  | ✔ |  |  |  | ✔ |  |  |  |  | ✔ |  |  |  |
| 2521 | 634 | CS |  |  |  |  | ✔ |  | ✔ |  |  |  |  | ✔ |  |  |  |
| 2509 | 638 | CS |  | ✔ |  |  |  |  | ✔ |  |  |  |  | ✔ |  |  |  |
| 2518 | 649 | CS |  |  | ✔ |  |  |  | ✔ |  |  |  |  | ✔ |  |  |  |
| 2507 | 650 | CS |  | NA | NA | NA | NA |  | NA | NA | NA | NA |  | ✔ |  |  |  |
| 2519 | 671 | CS |  |  |  | ✔ |  |  | ✔ |  |  |  |  | ✔ |  |  |  |
| 2522 | 673 | CS |  | ✔ |  |  |  |  | ✔ |  |  |  |  | ✔ |  |  |  |
| 2506 | 827 | CS |  |  |  |  | ✔ |  |  |  | ✔ |  |  | ✔ |  |  |  |
| 2513 | 905 | CS |  | ✔ |  |  |  |  | ✔ |  |  |  |  | ✔ |  |  |  |
| 2512 | 920 | CS |  |  |  |  | ✔ |  | ✔ |  |  |  |  | ✔ |  |  |  |
| 3518 | 720 | RB |  |  | ✔ |  |  |  |  |  | ✔ |  |  | ✔ |  |  |  |
| 3529 | 810 | RB |  |  | ✔ |  |  |  |  | ✔ |  |  |  |  |  |  |  |
| 3523 | 835 | RB |  | NA | NA | NA | NA |  | NA | NA | NA | NA |  | ✔ |  |  |  |
| 3543 | 870 | RB |  |  | ✔ |  |  |  |  | ✔ |  |  |  |  | ✔ |  |  |
| 3524 | 895 | RB |  |  | ✔ |  |  |  |  | ✔ |  |  |  | ✔ |  |  |  |
| 3534 | 899 | RB |  |  | ✔ |  |  |  |  | ✔ |  |  |  |  | ✔ |  |  |
| 3519 | 910 | RB |  |  |  | ✔ |  |  |  | ✔ |  |  |  | ✔ |  |  |  |
| 3536 | 930 | RB |  |  | ✔ |  |  |  |  | ✔ |  |  |  |  | ✔ |  |  |
| 3172 | 975 | RB |  |  | ✔ |  |  |  |  |  | ✔ |  |  |  | ✔ |  |  |
| 3535 | 980 | RB |  | ✔ |  |  |  |  |  |  | ✔ |  |  |  | ✔ |  |  |
| 3541 | 984 | RB |  |  | ✔ |  |  |  |  | ✔ |  |  |  |  | ✔ |  |  |
| 3537 | 988 | RB |  |  | ✔ |  |  |  |  | ✔ |  |  |  |  | ✔ |  |  |
| 3174 | 1000 | RB |  |  |  | ✔ |  |  |  | ✔ |  |  |  |  | ✔ |  |  |
| 3169 | 1005 | RB |  |  |  |  | ✔ |  |  | ✔ |  |  |  |  | ✔ |  |  |
| 3168 | 1009 | RB |  |  | ✔ |  |  |  |  | ✔ |  |  |  |  | ✔ |  |  |
| 3170 | 1010 | RB |  |  | ✔ |  |  |  |  | ✔ |  |  |  |  | ✔ |  |  |
| 3542 | 1012 | RB |  |  |  |  | ✔ |  |  | ✔ |  |  |  |  | ✔ |  |  |
| 3545 | 1019 | RB |  |  |  |  |  |  | ✔ |  |  |  |  |  | ✔ |  |  |
| 3530 | 1030 | RB |  |  | ✔ |  |  |  | ✔ |  |  |  |  |  | ✔ |  |  |
| 3525 | 1035 | RB |  |  |  | ✔ |  |  |  | ✔ |  |  |  |  | ✔ |  |  |
| 3167 | 1040 | RB |  | ✔ |  |  |  |  |  | ✔ |  |  |  |  | ✔ |  |  |
| 3520 | 1050 | RB |  |  | ✔ |  |  |  |  | ✔ |  |  |  |  | ✔ |  |  |
| 3171 | 1055 | RB |  |  | ✔ |  |  |  |  |  | ✔ |  |  |  | ✔ |  |  |
| 3521 | 1057 | RB |  | NA | NA | NA | NA |  | NA | NA | NA | NA |  |  | ✔ |  |  |
| 3526 | 1090 | RB |  |  |  | ✔ |  |  |  | ✔ |  |  |  |  | ✔ |  |  |
| 3531 | 1095 | RB |  |  |  | ✔ |  |  |  | ✔ |  |  |  |  | ✔ |  |  |
| 3173 | 1100 | RB |  |  |  | ✔ |  |  |  | ✔ |  |  |  |  | ✔ |  |  |
| 3532 | 1100 | RB |  |  |  |  | ✔ |  |  |  | ✔ |  |  |  | ✔ |  |  |
| 3522 | 1104 | RB |  |  | ✔ |  |  |  |  | ✔ |  |  |  |  | ✔ |  |  |
| 3539 | 1114 | RB |  | ✔ |  |  |  |  |  |  | ✔ |  |  |  | ✔ |  |  |
| 3527 | 1135 | RB |  |  | ✔ |  |  |  |  | ✔ |  |  |  |  | ✔ |  |  |
| 3528 | 1136 | RB |  |  | ✔ |  |  |  |  | ✔ |  |  |  |  | ✔ |  |  |
| 3540 | 1140 | RB |  |  |  |  | ✔ |  |  |  | ✔ |  |  |  | ✔ |  |  |
| 3544 | 1152 | RB |  | ✔ |  |  |  |  |  | ✔ |  |  |  |  | ✔ |  |  |
| 3533 | 1182 | RB |  |  |  |  | ✔ |  |  | ✔ |  |  |  |  | ✔ |  |  |
| 3538 | 1199 | RB |  |  |  |  | ✔ |  |  | ✔ |  |  |  |  | ✔ |  |  |
| 2736 | 540 | Wa |  |  |  | ✔ |  |  |  |  | ✔ |  |  |  |  | ✔ |  |
| 2737 | 583 | Wa |  |  | ✔ |  |  |  |  |  | ✔ |  |  | ✔ |  |  |  |
| 2734 | 600 | Wa |  |  |  |  | ✔ |  | ✔ |  |  |  |  |  |  | ✔ |  |
| 2744 | 605 | Wa |  |  | ✔ |  |  |  |  | ✔ |  |  |  |  |  | ✔ |  |
| 2976 | 610 | Wa |  |  | ✔ |  |  |  | ✔ |  |  |  |  | ✔ |  |  |  |
| 2735 | 620 | Wa |  |  |  | ✔ |  |  |  |  | ✔ |  |  |  |  | ✔ |  |
| 2739 | 625 | Wa |  |  |  |  | ✔ |  |  | ✔ |  |  |  |  |  | ✔ |  |
| 2738 | 630 | Wa |  |  | ✔ |  |  |  |  |  | ✔ |  |  |  |  | ✔ |  |
| 2742 | 630 | Wa |  |  | ✔ |  |  |  |  |  | ✔ |  |  |  |  | ✔ |  |
| 2746 | 670 | Wa |  |  |  | ✔ |  |  |  | ✔ |  |  |  |  |  | ✔ |  |
| 2740 | 675 | Wa |  |  |  | ✔ |  |  |  |  | ✔ |  |  |  |  | ✔ |  |
| 2743 | 685 | Wa |  |  |  |  | ✔ |  |  |  | ✔ |  |  |  |  | ✔ |  |
| 2747 | 710 | Wa |  |  |  | ✔ |  |  | ✔ |  |  |  |  |  |  | ✔ |  |
| 2748 | 720 | Wa |  |  |  | ✔ |  |  |  |  | ✔ |  |  |  |  | ✔ |  |
| 2741 | 730 | Wa |  |  |  | ✔ |  |  |  |  | ✔ |  |  |  |  |  | ✔ |
| 2745 | 735 | Wa |  |  | ✔ |  |  |  |  |  | ✔ |  |  |  |  | ✔ |  |
| 2749 | 813 | Wa |  | NA | NA | NA | NA |  | NA | NA | NA | NA |  |  |  | ✔ |  |
| 2974 | 885 | Wa |  |  |  |  | ✔ |  |  | ✔ |  |  |  |  | ✔ |  |  |
| 2975 | 1040 | Wa |  |  |  | ✔ |  |  |  |  | ✔ |  |  |  |  | ✔ |  |
| 2971 | 1085 | Wa |  |  |  | ✔ |  |  |  |  | ✔ |  |  |  |  |  | ✔ |
| 2968 | 1090 | Wa |  |  | ✔ |  |  |  |  |  | ✔ |  |  |  |  | ✔ |  |
| 2969 | 1090 | Wa |  |  | ✔ |  |  |  |  |  | ✔ |  |  |  |  | ✔ |  |
| 2973 | 1090 | Wa |  |  |  |  | ✔ |  | ✔ |  |  |  |  | ✔ |  |  |  |
| 2970 | 1110 | Wa |  |  | ✔ |  |  |  | ✔ |  |  |  |  |  |  | ✔ |  |
| 2972 | 1160 | Wa |  |  |  | ✔ |  |  |  | ✔ |  |  |  |  |  |  | ✔ |
| 3570 | 804 | Wy |  |  |  |  | ✔ |  |  |  |  | ✔ |  | ✔ |  |  |  |
| 3557 | 822 | Wy |  |  |  |  | ✔ |  |  |  |  | ✔ |  | ✔ |  |  |  |
| 3548 | 860 | Wy |  |  |  |  | ✔ |  |  |  |  | ✔ |  |  |  |  | ✔ |
| 3547 | 865 | Wy |  | NA | NA | NA | NA |  | NA | NA | NA | NA |  | ✔ |  |  |  |
| 3564 | 871 | Wy |  |  |  |  | ✔ |  |  |  |  | ✔ |  |  |  | ✔ |  |
| 3558 | 873 | Wy |  |  |  |  | ✔ |  |  |  |  | ✔ |  | ✔ |  |  |  |
| 3567 | 882 | Wy |  |  |  |  | ✔ |  |  |  | ✔ |  |  |  |  |  | ✔ |
| 3560 | 895 | Wy |  |  |  |  | ✔ |  |  |  |  | ✔ |  |  |  |  | ✔ |
| 3556 | 920 | Wy |  |  |  |  | ✔ |  |  |  |  | ✔ |  |  |  |  | ✔ |
| 3566 | 935 | Wy |  |  | ✔ |  |  |  |  |  |  | ✔ |  |  |  |  | ✔ |
| 3573 | 940 | Wy |  |  |  |  | ✔ |  | ✔ |  |  |  |  |  |  |  | ✔ |
| 3574 | 946 | Wy |  |  |  |  | ✔ |  |  |  |  | ✔ |  |  |  | ✔ |  |
| 3569 | 1085 | Wy |  |  | ✔ |  |  |  | ✔ |  |  |  |  | ✔ |  |  |  |
| 3572 | 1112 | Wy |  |  | ✔ |  |  |  | ✔ |  |  |  |  |  |  |  | ✔ |
| 3555 | 1114 | Wy |  |  |  |  | ✔ |  |  |  |  | ✔ |  |  |  | ✔ |  |
| 3578 | 1114 | Wy |  |  |  |  | ✔ |  |  |  |  | ✔ |  |  |  | ✔ |  |
| 3554 | 1128 | Wy |  |  |  |  | ✔ |  |  |  |  | ✔ |  |  |  | ✔ |  |
| 3563 | 1132 | Wy |  |  | ✔ |  |  |  |  |  |  | ✔ |  |  |  |  | ✔ |
| 3571 | 1132 | Wy |  |  |  |  | ✔ |  |  |  |  | ✔ |  |  |  |  | ✔ |
| 3579 | 1140 | Wy |  |  |  | ✔ |  |  |  |  | ✔ |  |  |  |  | ✔ |  |
| 3576 | 1154 | Wy |  |  |  | ✔ |  |  |  | ✔ |  |  |  |  |  |  | ✔ |
| 3559 | 1155 | Wy |  |  |  |  | ✔ |  |  |  |  | ✔ |  |  |  |  | ✔ |
| 3552 | 1168 | Wy |  |  |  |  | ✔ |  |  |  |  | ✔ |  |  |  |  | ✔ |
| 3550 | 1171 | Wy |  |  |  |  | ✔ |  |  |  | ✔ |  |  |  |  |  | ✔ |
| 3551 | 1171 | Wy |  |  |  |  | ✔ |  |  |  |  | ✔ |  |  |  |  | ✔ |
| 3575 | 1175 | Wy |  |  |  |  | ✔ |  |  |  |  | ✔ |  |  |  |  | ✔ |
| 3577 | 1188 | Wy |  |  |  |  | ✔ |  |  |  |  | ✔ |  |  |  |  | ✔ |
| 3546 | 1191 | Wy |  | NA | NA | NA | NA |  | NA | NA | NA | NA |  |  |  |  | ✔ |
| 3549 | 1195 | Wy |  |  | ✔ |  |  |  |  |  |  | ✔ |  |  |  |  | ✔ |
| 3553 | 1200 | Wy |  |  |  |  | ✔ |  |  |  |  | ✔ |  |  |  |  | ✔ |
| 3565 | 1210 | Wy |  | NA | NA | NA | NA |  | NA | NA | NA | NA |  | ✔ |  |  |  |
| 3561 | 1215 | Wy |  |  |  | ✔ |  |  |  |  |  | ✔ |  |  |  |  | ✔ |
| 3568 | 1220 | Wy |  |  |  |  | ✔ |  |  |  |  | ✔ |  |  |  |  | ✔ |
| 3562 | 1300 | Wy |  | NA | NA | NA | NA |  | NA | NA | NA | NA |  |  |  |  | ✔ |

**b)**

|  |  |  |  | **Assigned Location** | | | | | | | | | | | | | | | | |
| --- | --- | --- | --- | --- | --- | --- | --- | --- | --- | --- | --- | --- | --- | --- | --- | --- | --- | --- | --- | --- |
|  |  |  |  | **Otolith Near Core** | | | | |  | **Otolith Margin** | | | | |  | **Parasites** | | | | |
| **Sample** | **Size (mm)** | **Sampling location** |  | **BI** | **MI** | **OD** | **PI** | **Wa** |  | **BI** | **MI** | **OD** | **PI** | **Wa** |  | **BI** | **MI** | **OD** | **PI** | **Wa** |
| 2272 | 1120 | BI |  |  | ✔ |  |  |  |  | ✔ |  |  |  |  |  |  |  |  | ✔ |  |
| 2273 | 1160 | BI |  |  |  |  |  | ✔ |  | ✔ |  |  |  |  |  | ✔ |  |  |  |  |
| 2282 | 1215 | BI |  |  | ✔ |  |  |  |  |  |  |  |  | ✔ |  | ✔ |  |  |  |  |
| 2921 | 550 | BI |  | ✔ |  |  |  |  |  | ✔ |  |  |  |  |  |  |  | ✔ |  |  |
| 2922 | 590 | BI |  |  | ✔ |  |  |  |  | ✔ |  |  |  |  |  |  |  | ✔ |  |  |
| 3023 | 1130 | BI |  |  | ✔ |  |  |  |  |  |  |  | ✔ |  |  |  |  |  | ✔ |  |
| 3024 | 1120 | BI |  | ✔ |  |  |  |  |  | ✔ |  |  |  |  |  |  |  |  |  | ✔ |
| 3025 | 1115 | BI |  | ✔ |  |  |  |  |  | ✔ |  |  |  |  |  |  |  |  |  | ✔ |
| 3026 | 1150 | BI |  | ✔ |  |  |  |  |  |  |  |  |  | ✔ |  | ✔ |  |  |  |  |
| 3027 | 1235 | BI |  | NA | NA | NA | NA | NA |  | NA | NA | NA | NA | NA |  |  |  |  |  | ✔ |
| 3028 | 1015 | BI |  | ✔ |  |  |  |  |  |  |  |  | ✔ |  |  |  |  |  |  | ✔ |
| 3029 | 387 | BI |  | ✔ |  |  |  |  |  |  |  |  |  | ✔ |  |  |  | ✔ |  |  |
| 3030 | 1170 | BI |  |  | ✔ |  |  |  |  |  | ✔ |  |  |  |  |  |  |  |  | ✔ |
| 3031 | 1165 | BI |  | ✔ |  |  |  |  |  | ✔ |  |  |  |  |  | ✔ |  |  |  |  |
| 3032 | 1120 | BI |  |  | ✔ |  |  |  |  | ✔ |  |  |  |  |  |  |  |  |  | ✔ |
| 3466 | 1050 | BI |  |  |  |  |  | ✔ |  | ✔ |  |  |  |  |  | ✔ |  |  |  |  |
| 3467 | 1090 | BI |  |  |  |  | ✔ |  |  | ✔ |  |  |  |  |  |  |  |  |  | ✔ |
| 3468 | 510 | BI |  | ✔ |  |  |  |  |  |  |  |  | ✔ |  |  |  |  | ✔ |  |  |
| 3469 | 520 | BI |  | ✔ |  |  |  |  |  |  | ✔ |  |  |  |  |  |  | ✔ |  |  |
| 3509 | 1100 | BI |  | ✔ |  |  |  |  |  | ✔ |  |  |  |  |  | ✔ |  |  |  |  |
| 3510 |  | BI |  |  | ✔ |  |  |  |  | ✔ |  |  |  |  |  | ✔ |  |  |  |  |
| 3511 | 1080 | BI |  |  |  | ✔ |  |  |  | ✔ |  |  |  |  |  | ✔ |  |  |  |  |
| 3512 |  | BI |  | ✔ |  |  |  |  |  | ✔ |  |  |  |  |  | ✔ |  |  |  |  |
| 3513 |  | BI |  | ✔ |  |  |  |  |  | ✔ |  |  |  |  |  | ✔ |  |  |  |  |
| 3514 |  | BI |  |  |  |  |  | ✔ |  | ✔ |  |  |  |  |  | ✔ |  |  |  |  |
| 3515 |  | BI |  |  |  |  | ✔ |  |  |  |  |  | ✔ |  |  | ✔ |  |  |  |  |
| 3516 |  | BI |  | ✔ |  |  |  |  |  | ✔ |  |  |  |  |  | ✔ |  |  |  |  |
| 3517 |  | BI |  |  | ✔ |  |  |  |  |  |  |  |  | ✔ |  | ✔ |  |  |  |  |
| 1482 | 445 | MI |  | ✔ |  |  |  |  |  |  |  |  |  | ✔ |  |  |  | ✔ |  |  |
| 2178 | 540 | MI |  |  |  |  |  |  |  |  |  | ✔ |  |  |  |  | ✔ |  |  |  |
| 2179 | 605 | MI |  | ✔ |  |  |  |  |  |  |  | ✔ |  |  |  |  | ✔ |  |  |  |
| 3222 | 1060 | MI |  | ✔ |  |  |  |  |  |  | ✔ |  |  |  |  | ✔ |  |  |  |  |
| 3223 | 1170 | MI |  |  |  |  | ✔ |  |  |  | ✔ |  |  |  |  | ✔ |  |  |  |  |
| 3224 | 770 | MI |  |  | ✔ |  |  |  |  |  |  |  |  | ✔ |  |  | ✔ |  |  |  |
| 3225 | 735 | MI |  |  | ✔ |  |  |  |  |  | ✔ |  |  |  |  |  | ✔ |  |  |  |
| 3226 | 415 | MI |  |  | ✔ |  |  |  |  |  |  |  | ✔ |  |  |  |  |  |  | ✔ |
| 3227 | 405 | MI |  |  |  |  |  | ✔ |  |  |  |  |  |  |  |  | ✔ |  |  |  |
| 3228 | 405 | MI |  |  | ✔ |  |  |  |  |  | ✔ |  |  |  |  |  | ✔ |  |  |  |
| 3446 | 1020 | MI |  |  | ✔ |  |  |  |  | ✔ |  |  |  |  |  | ✔ |  |  |  |  |
| 3447 | 480 | MI |  |  | ✔ |  |  |  |  |  | ✔ |  |  |  |  |  | ✔ |  |  |  |
| 3448 | 560 | MI |  |  |  |  | ✔ |  |  |  | ✔ |  |  |  |  |  |  | ✔ |  |  |
| 3449 | 790 | MI |  |  | ✔ |  |  |  |  |  | ✔ |  |  |  |  |  | ✔ |  |  |  |
| 3450 | 1010 | MI |  |  | ✔ |  |  |  |  |  |  |  |  | ✔ |  | ✔ |  |  |  |  |
| 3451 | 1060 | MI |  |  |  |  | ✔ |  |  |  | ✔ |  |  |  |  |  |  |  | ✔ |  |
| 3452 | 710 | MI |  | NA | NA | NA | NA | NA |  | NA | NA | NA | NA | NA |  |  | ✔ |  |  |  |
| 3453 | 530 | MI |  |  | ✔ |  |  |  |  |  | ✔ |  |  |  |  |  | ✔ |  |  |  |
| 3454 | 610 | MI |  | ✔ |  |  |  |  |  |  | ✔ |  |  |  |  |  |  |  |  | ✔ |
| 3455 | 640 | MI |  |  | ✔ |  |  |  |  |  | ✔ |  |  |  |  |  | ✔ |  |  |  |
| 3456 | 560 | MI |  |  |  |  |  | ✔ |  |  |  |  |  |  |  |  | ✔ |  |  |  |
| 3457 | 530 | MI |  |  | ✔ |  |  |  |  |  |  |  | ✔ |  |  |  |  | ✔ |  |  |
| 3458 | 540 | MI |  |  |  |  | ✔ |  |  | ✔ |  |  |  |  |  |  |  | ✔ |  |  |
| 3459 | 700 | MI |  |  |  |  | ✔ |  |  |  | ✔ |  |  |  |  |  |  |  |  | ✔ |
| 3460 | 710 | MI |  |  |  |  | ✔ |  |  |  | ✔ |  |  |  |  |  |  |  | ✔ |  |
| 3461 | 520 | MI |  |  |  |  | ✔ |  |  |  | ✔ |  |  |  |  |  | ✔ |  |  |  |
| 3462 | 480 | MI |  | ✔ |  |  |  |  |  |  | ✔ |  |  |  |  |  | ✔ |  |  |  |
| 3463 | 470 | MI |  | ✔ |  |  |  |  |  |  |  | ✔ |  |  |  |  |  | ✔ |  |  |
| 3464 | 420 | MI |  |  |  |  | ✔ |  |  |  | ✔ |  |  |  |  |  |  | ✔ |  |  |
| 3465 | 500 | MI |  |  | ✔ |  |  |  |  |  |  |  | ✔ |  |  |  |  | ✔ |  |  |
| 1705 | 610 | OD |  | ✔ |  |  |  |  |  |  |  |  |  | ✔ |  |  |  | ✔ |  |  |
| 1709 | 565 | OD |  |  |  | ✔ |  |  |  |  | ✔ |  |  |  |  |  |  | ✔ |  |  |
| 1710 | 550 | OD |  | ✔ |  |  |  |  |  |  | ✔ |  |  |  |  |  |  | ✔ |  |  |
| 1711 | 460 | OD |  |  | ✔ |  |  |  |  |  |  |  |  | ✔ |  |  |  | ✔ |  |  |
| 1712 | 450 | OD |  |  |  |  | ✔ |  |  |  | ✔ |  |  |  |  |  |  | ✔ |  |  |
| 1714 | 405 | OD |  |  |  |  | ✔ |  |  |  | ✔ |  |  |  |  |  |  | ✔ |  |  |
| 1715 | 500 | OD |  |  |  |  | ✔ |  |  |  | ✔ |  |  |  |  |  |  | ✔ |  |  |
| 1919 | 890 | OD |  |  |  |  | ✔ |  |  |  |  |  | ✔ |  |  |  |  |  |  | ✔ |
| 1920 | 980 | OD |  | ✔ |  |  |  |  |  |  | ✔ |  |  |  |  |  |  | ✔ |  |  |
| 1921 | 1150 | OD |  |  |  |  |  | ✔ |  |  | ✔ |  |  |  |  |  |  | ✔ |  |  |
| 1922 | 930 | OD |  |  |  |  | ✔ |  |  |  | ✔ |  |  |  |  |  | ✔ |  |  |  |
| 2194 | 540 | OD |  |  |  | ✔ |  |  |  |  |  | ✔ |  |  |  |  |  | ✔ |  |  |
| 2221 | 460 | OD |  |  |  | ✔ |  |  |  |  | ✔ |  |  |  |  |  | ✔ |  |  |  |
| 2222 | 395 | OD |  |  | ✔ |  |  |  |  |  |  |  |  | ✔ |  |  |  | ✔ |  |  |
| 2223 | 405 | OD |  |  | ✔ |  |  |  |  | ✔ |  |  |  |  |  |  |  | ✔ |  |  |
| 2224 | 395 | OD |  |  |  |  |  | ✔ |  |  |  | ✔ |  |  |  |  |  | ✔ |  |  |
| 2598 | 650 | OD |  |  |  |  |  | ✔ |  |  |  |  |  | ✔ |  |  |  |  | ✔ |  |
| 3259 |  | PI |  |  |  |  |  | ✔ |  |  |  |  | ✔ |  |  | ✔ |  |  |  |  |
| 3260 |  | PI |  |  | ✔ |  |  |  |  |  | ✔ |  |  |  |  | NA | NA | NA | NA | NA |
| 3261 |  | PI |  | ✔ |  |  |  |  |  | ✔ |  |  |  |  |  | ✔ |  |  |  |  |
| 3262 |  | PI |  |  |  |  | ✔ |  |  |  |  |  | ✔ |  |  |  |  |  | ✔ |  |
| 3263 |  | PI |  |  |  |  | ✔ |  |  |  |  |  | ✔ |  |  | ✔ |  |  |  |  |
| 3264 |  | PI |  | ✔ |  |  |  |  |  |  |  |  | ✔ |  |  | NA | NA | NA | NA | NA |
| 3265 |  | PI |  | ✔ |  |  |  |  |  |  |  |  | ✔ |  |  |  |  |  |  | ✔ |
| 3266 |  | PI |  |  | ✔ |  |  |  |  |  |  |  | ✔ |  |  | NA | NA | NA | NA | NA |
| 3267 |  | PI |  |  |  |  | ✔ |  |  |  |  |  | ✔ |  |  | NA | NA | NA | NA | NA |
| 3268 |  | PI |  |  |  |  | ✔ |  |  |  |  |  | ✔ |  |  |  |  |  | ✔ |  |
| 3269 |  | PI |  |  |  |  | ✔ |  |  |  |  |  | ✔ |  |  | NA | NA | NA | NA | NA |
| 3270 |  | PI |  |  |  |  | ✔ |  |  |  |  |  | ✔ |  |  |  |  |  | ✔ |  |
| 3271 |  | PI |  |  |  |  | ✔ |  |  |  |  |  | ✔ |  |  |  |  |  | ✔ |  |
| 3272 |  | PI |  |  |  |  | ✔ |  |  |  |  |  | ✔ |  |  | NA | NA | NA | NA | NA |
| 3273 |  | PI |  |  |  |  | ✔ |  |  |  |  |  | ✔ |  |  | NA | NA | NA | NA | NA |
| 3274 |  | PI |  |  |  |  |  | ✔ |  |  |  |  | ✔ |  |  |  |  | ✔ |  |  |
| 3275 |  | PI |  |  |  |  | ✔ |  |  |  |  |  | ✔ |  |  |  |  |  | ✔ |  |
| 3276 |  | PI |  |  |  |  | ✔ |  |  |  |  |  | ✔ |  |  |  |  |  | ✔ |  |
| 3277 |  | PI |  |  | ✔ |  |  |  |  |  |  |  | ✔ |  |  |  |  |  | ✔ |  |
| 3278 |  | PI |  |  | ✔ |  |  |  |  |  |  |  | ✔ |  |  |  |  |  | ✔ |  |
| 3279 |  | PI |  |  |  |  | ✔ |  |  |  |  |  | ✔ |  |  |  |  |  | ✔ |  |
| 3280 |  | PI |  |  |  |  | ✔ |  |  |  |  |  | ✔ |  |  |  |  |  | ✔ |  |
| 3281 |  | PI |  |  |  |  | ✔ |  |  |  |  |  | ✔ |  |  |  |  |  |  | ✔ |
| 3282 |  | PI |  | ✔ |  |  |  |  |  |  |  |  | ✔ |  |  |  |  |  |  | ✔ |
| 3283 |  | PI |  |  |  | ✔ |  |  |  |  |  |  | ✔ |  |  |  |  |  | ✔ |  |
| 3284 |  | PI |  |  |  |  | ✔ |  |  |  |  |  | ✔ |  |  |  |  |  | ✔ |  |
| 3285 |  | PI |  |  |  |  | ✔ |  |  |  |  |  | ✔ |  |  |  |  |  | ✔ |  |
| 3286 |  | PI |  |  |  |  | ✔ |  |  |  |  |  | ✔ |  |  |  |  |  | ✔ |  |
| 3287 |  | PI |  |  |  |  | ✔ |  |  |  |  |  | ✔ |  |  |  |  |  | ✔ |  |
| 2734 | 600 | Wa |  |  |  |  |  | ✔ |  |  |  |  |  | ✔ |  |  |  |  |  | ✔ |
| 2735 | 620 | Wa |  |  |  |  |  | ✔ |  |  |  |  |  | ✔ |  |  | ✔ |  |  |  |
| 2736 | 540 | Wa |  |  |  |  |  | ✔ |  |  | ✔ |  |  |  |  |  | ✔ |  |  |  |
| 2737 | 583 | Wa |  |  | ✔ |  |  |  |  |  |  |  |  | ✔ |  |  | ✔ |  |  |  |
| 2738 | 630 | Wa |  |  | ✔ |  |  |  |  |  | ✔ |  |  |  |  |  |  |  |  | ✔ |
| 2739 | 625 | Wa |  |  |  | ✔ |  |  |  |  |  |  |  | ✔ |  |  |  |  |  | ✔ |
| 2740 | 675 | Wa |  |  |  | ✔ |  |  |  |  |  |  | ✔ |  |  |  |  |  |  | ✔ |
| 2741 | 730 | Wa |  |  |  | ✔ |  |  |  |  |  |  |  | ✔ |  | ✔ |  |  |  |  |
| 2742 | 630 | Wa |  |  | ✔ |  |  |  |  |  | ✔ |  |  |  |  |  |  |  |  | ✔ |
| 2743 | 685 | Wa |  | ✔ |  |  |  |  |  |  |  |  |  | ✔ |  |  |  |  |  | ✔ |
| 2744 | 605 | Wa |  | ✔ |  |  |  |  |  |  |  |  |  | ✔ |  |  |  |  |  | ✔ |
| 2745 | 735 | Wa |  | ✔ |  |  |  |  |  |  |  |  | ✔ |  |  |  |  |  |  | ✔ |
| 2746 | 670 | Wa |  |  |  |  |  | ✔ |  | ✔ |  |  |  |  |  |  |  |  |  | ✔ |
| 2747 | 710 | Wa |  |  |  |  |  | ✔ |  |  |  | ✔ |  |  |  |  |  |  |  | ✔ |
| 2748 | 720 | Wa |  |  |  |  |  | ✔ |  |  |  | ✔ |  |  |  |  |  | ✔ |  |  |
| 2749 | 813 | Wa |  | NA | NA | NA | NA | NA |  | NA | NA | NA | NA | NA |  |  |  |  |  | ✔ |
| 2968 | 1090 | Wa |  |  | ✔ |  |  |  |  |  |  | ✔ |  |  |  |  |  |  | ✔ |  |
| 2969 | 1090 | Wa |  |  | ✔ |  |  |  |  |  |  |  | ✔ |  |  | ✔ |  |  |  |  |
| 2970 | 1110 | Wa |  | ✔ |  |  |  |  |  |  | ✔ |  |  |  |  |  |  |  |  | ✔ |
| 2971 | 1085 | Wa |  |  |  |  | ✔ |  |  | ✔ |  |  |  |  |  | ✔ |  |  |  |  |
| 2972 | 1160 | Wa |  |  |  |  |  | ✔ |  | ✔ |  |  |  |  |  |  |  |  | ✔ |  |
| 2973 | 1090 | Wa |  |  |  |  | ✔ |  |  | ✔ |  |  |  |  |  |  |  |  | ✔ |  |
| 2974 | 885 | Wa |  |  |  |  |  | ✔ |  | ✔ |  |  |  |  |  |  | ✔ |  |  |  |
| 2975 | 1040 | Wa |  |  |  |  |  | ✔ |  |  |  |  |  | ✔ |  | ✔ |  |  |  |  |
| 2976 | 610 | Wa |  |  | ✔ |  |  |  |  |  |  |  | ✔ |  |  |  |  | ✔ |  |  |

**c)**

|  |  |  |  | **Assigned Location** | | | | | | | | | | | | | |
| --- | --- | --- | --- | --- | --- | --- | --- | --- | --- | --- | --- | --- | --- | --- | --- | --- | --- |
|  |  |  |  | **Otolith Near Core** | | | |  | **Otolith Margin** | | | |  | **Parasites** | | | |
| **Sample** | **Size (mm)** | **Sampling location** |  | **AS** | **Ma** | **MI** | **VI** |  | **AS** | **Ma** | **MI** | **VI** |  | **AS** | **Ma** | **MI** | **VI** |
| 1960 | NA | AS |  | ✔ |  |  |  |  | ✔ |  |  |  |  | ✔ |  |  |  |
| 1963 | NA | AS |  | ✔ |  |  |  |  | ✔ |  |  |  |  | ✔ |  |  |  |
| 1964 | NA | AS |  | ✔ |  |  |  |  | ✔ |  |  |  |  | ✔ |  |  |  |
| 1973 | NA | AS |  | ✔ |  |  |  |  | ✔ |  |  |  |  | ✔ |  |  |  |
| 1956 | NA | AS |  |  | ✔ |  |  |  | ✔ |  |  |  |  | ✔ |  |  |  |
| 1958 | NA | AS |  |  | ✔ |  |  |  | ✔ |  |  |  |  | ✔ |  |  |  |
| 1959 | NA | AS |  |  | ✔ |  |  |  | ✔ |  |  |  |  | ✔ |  |  |  |
| 1965 | NA | AS |  |  | ✔ |  |  |  | ✔ |  |  |  |  | ✔ |  |  |  |
| 1968 | NA | AS |  |  | ✔ |  |  |  | ✔ |  |  |  |  | ✔ |  |  |  |
| 1970 | NA | AS |  |  | ✔ |  |  |  | ✔ |  |  |  |  | ✔ |  |  |  |
| 1957 | NA | AS |  |  |  |  | ✔ |  | ✔ |  |  |  |  | ✔ |  |  |  |
| 1966 | NA | AS |  |  |  |  | ✔ |  | ✔ |  |  |  |  | ✔ |  |  |  |
| 1967 | NA | AS |  |  |  |  | ✔ |  | ✔ |  |  |  |  | ✔ |  |  |  |
| 1971 | NA | AS |  |  |  |  | ✔ |  | ✔ |  |  |  |  | ✔ |  |  |  |
| 1972 | NA | AS |  |  |  |  | ✔ |  | ✔ |  |  |  |  | ✔ |  |  |  |
| 1974 | NA | AS |  |  |  |  | ✔ |  | ✔ |  |  |  |  | ✔ |  |  |  |
| 1969 | NA | AS |  | ✔ |  |  |  |  |  | ✔ |  |  |  | ✔ |  |  |  |
| 1961 | NA | AS |  |  |  | ✔ |  |  | ✔ |  |  |  |  |  |  | ✔ |  |
| 1962 | NA | AS |  |  |  |  | ✔ |  | ✔ |  |  |  |  |  |  | ✔ |  |
| 1955 | NA | AS |  |  | ✔ |  |  |  | ✔ |  |  |  |  |  |  |  | ✔ |
| 2812 | 420 | Ma |  |  |  |  | ✔ |  |  | ✔ |  |  |  |  | ✔ |  |  |
| 2827 | 460 | Ma |  |  |  | ✔ |  |  |  | ✔ |  |  |  |  | ✔ |  |  |
| 2806 | 510 | Ma |  |  | ✔ |  |  |  |  | ✔ |  |  |  |  | ✔ |  |  |
| 2822 | 510 | Ma |  |  |  | ✔ |  |  |  |  |  | ✔ |  |  | ✔ |  |  |
| 2828 | 520 | Ma |  | ✔ |  |  |  |  |  |  | ✔ |  |  |  | ✔ |  |  |
| 2811 | 520 | Ma |  |  | ✔ |  |  |  |  |  | ✔ |  |  |  | ✔ |  |  |
| 2813 | 530 | Ma |  |  |  |  | ✔ |  |  | ✔ |  |  |  |  | ✔ |  |  |
| 2824 | 530 | Ma |  | ✔ |  |  |  |  |  |  | ✔ |  |  |  | ✔ |  |  |
| 2826 | 540 | Ma |  |  |  | ✔ |  |  |  | ✔ |  |  |  |  | ✔ |  |  |
| 2807 | 540 | Ma |  |  |  |  | ✔ |  |  |  |  | ✔ |  |  | ✔ |  |  |
| 2816 | 560 | Ma |  |  | ✔ |  |  |  |  | ✔ |  |  |  |  | ✔ |  |  |
| 2808 | 570 | Ma |  |  | ✔ |  |  |  |  | ✔ |  |  |  |  | ✔ |  |  |
| 2810 | 590 | Ma |  |  | ✔ |  |  |  |  | ✔ |  |  |  |  | ✔ |  |  |
| 2825 | 600 | Ma |  |  |  | ✔ |  |  | ✔ |  |  |  |  |  | ✔ |  |  |
| 2809 | 620 | Ma |  |  | ✔ |  |  |  |  |  | ✔ |  |  |  | ✔ |  |  |
| 2815 | 710 | Ma |  |  |  | ✔ |  |  |  | ✔ |  |  |  |  | ✔ |  |  |
| 2818 | 830 | Ma |  |  |  | ✔ |  |  |  | ✔ |  |  |  |  | ✔ |  |  |
| 3362 | 840 | Ma |  |  |  | ✔ |  |  |  |  |  | ✔ |  |  | ✔ |  |  |
| 3366 | 860 | Ma |  |  |  | ✔ |  |  |  |  |  | ✔ |  |  | ✔ |  |  |
| 3365 | 920 | Ma |  |  | ✔ |  |  |  |  | ✔ |  |  |  |  | ✔ |  |  |
| 3364 | 970 | Ma |  |  | ✔ |  |  |  |  | ✔ |  |  |  |  | ✔ |  |  |
| 3393 | 1190 | Ma |  |  | ✔ |  |  |  |  | ✔ |  |  |  |  | ✔ |  |  |
| 3396 | 1195 | Ma |  |  |  |  | ✔ |  |  |  | ✔ |  |  |  | ✔ |  |  |
| 3397 | 1200 | Ma |  |  |  |  | ✔ |  |  | ✔ |  |  |  |  | ✔ |  |  |
| 3394 | 1210 | Ma |  |  |  | ✔ |  |  |  |  | ✔ |  |  |  | ✔ |  |  |
| 3395 | NA | Ma |  | ✔ |  |  |  |  |  |  |  | ✔ |  |  | ✔ |  |  |
| 2817 | 680 | Ma |  |  | ✔ |  |  |  |  |  |  | ✔ |  |  |  | ✔ |  |
| 2823 | 590 | Ma |  |  |  | ✔ |  |  |  |  | ✔ |  |  |  |  |  | ✔ |
| 3363 | 810 | Ma |  |  | ✔ |  |  |  |  |  | ✔ |  |  |  |  |  | ✔ |
| 3398 | 1170 | Ma |  |  | ✔ |  |  |  | ✔ |  |  |  |  |  |  |  | ✔ |
| 3451 | 1060 | MI |  |  | ✔ |  |  |  |  |  | ✔ |  |  | ✔ |  |  |  |
| 3228 | 405 | MI |  |  |  | ✔ |  |  |  |  | ✔ |  |  |  |  | ✔ |  |
| 3226 | 415 | MI |  |  |  | ✔ |  |  |  |  | ✔ |  |  |  |  | ✔ |  |
| 3464 | 420 | MI |  |  |  | ✔ |  |  |  |  | ✔ |  |  |  |  | ✔ |  |
| 3462 | 480 | MI |  | ✔ |  |  |  |  |  |  | ✔ |  |  |  |  | ✔ |  |
| 3447 | 480 | MI |  |  | ✔ |  |  |  |  |  | ✔ |  |  |  |  | ✔ |  |
| 3465 | 500 | MI |  |  |  | ✔ |  |  |  |  | ✔ |  |  |  |  | ✔ |  |
| 3461 | 520 | MI |  |  |  |  | ✔ |  | ✔ |  |  |  |  |  |  | ✔ |  |
| 3457 | 530 | MI |  |  | ✔ |  |  |  |  |  | ✔ |  |  |  |  | ✔ |  |
| 3453 | 530 | MI |  |  |  | ✔ |  |  |  |  | ✔ |  |  |  |  | ✔ |  |
| 3458 | 540 | MI |  |  |  | ✔ |  |  |  |  | ✔ |  |  |  |  | ✔ |  |
| 3448 | 560 | MI |  | ✔ |  |  |  |  | ✔ |  |  |  |  |  |  | ✔ |  |
| 3456 | 560 | MI |  | ✔ |  |  |  |  |  |  |  | ✔ |  |  |  | ✔ |  |
| 3454 | 610 | MI |  |  |  |  | ✔ |  |  |  | ✔ |  |  |  |  | ✔ |  |
| 3455 | 640 | MI |  |  |  | ✔ |  |  |  |  | ✔ |  |  |  |  | ✔ |  |
| 3459 | 700 | MI |  |  |  |  | ✔ |  |  |  | ✔ |  |  |  |  | ✔ |  |
| 3460 | 710 | MI |  |  |  | ✔ |  |  |  |  | ✔ |  |  |  |  | ✔ |  |
| 3452 | 710 | MI |  | NA | NA | NA | NA |  | NA | NA | NA | NA |  |  |  | ✔ |  |
| 3225 | 735 | MI |  |  |  | ✔ |  |  | ✔ |  |  |  |  |  |  | ✔ |  |
| 3224 | 770 | MI |  |  |  | ✔ |  |  |  |  | ✔ |  |  |  |  | ✔ |  |
| 3449 | 790 | MI |  |  |  | ✔ |  |  |  |  | ✔ |  |  |  |  | ✔ |  |
| 3446 | 1020 | MI |  |  | ✔ |  |  |  |  |  | ✔ |  |  |  |  | ✔ |  |
| 3223 | 1170 | MI |  |  |  | ✔ |  |  | ✔ |  |  |  |  |  |  | ✔ |  |
| 3227 | 405 | MI |  |  | ✔ |  |  |  |  |  | ✔ |  |  |  |  |  | ✔ |
| 1482 | 445 | MI |  |  |  |  | ✔ |  |  | ✔ |  |  |  |  |  |  | ✔ |
| 3463 | 470 | MI |  |  |  |  | ✔ |  |  |  | ✔ |  |  |  |  |  | ✔ |
| 2178 | 540 | MI |  |  | ✔ |  |  |  |  |  | ✔ |  |  |  |  |  | ✔ |
| 2179 | 605 | MI |  | ✔ |  |  |  |  |  |  | ✔ |  |  |  |  |  | ✔ |
| 3450 | 1010 | MI |  |  | ✔ |  |  |  |  |  | ✔ |  |  |  |  |  | ✔ |
| 3222 | 1060 | MI |  |  | ✔ |  |  |  |  |  | ✔ |  |  |  |  |  | ✔ |
| 2547 | 440 | VI |  |  |  |  | ✔ |  |  |  |  | ✔ |  |  |  |  | ✔ |
| 2545 | 475 | VI |  |  |  |  | ✔ |  |  |  |  | ✔ |  |  |  |  | ✔ |
| 2550 | 485 | VI |  |  |  | ✔ |  |  |  |  |  | ✔ |  |  |  |  | ✔ |
| 2540 | 520 | VI |  |  |  |  | ✔ |  |  |  |  | ✔ |  |  |  |  | ✔ |
| 2544 | 530 | VI |  | NA | NA | NA | NA |  | NA | NA | NA | NA |  |  |  |  | ✔ |
| 2538 | 530 | VI |  |  |  |  | ✔ |  |  |  |  | ✔ |  |  |  |  | ✔ |
| 2541 | 530 | VI |  |  |  |  | ✔ |  |  |  |  | ✔ |  |  |  |  | ✔ |
| 2535 | 540 | VI |  |  |  |  | ✔ |  |  |  |  | ✔ |  |  |  |  | ✔ |
| 2543 | 550 | VI |  |  | ✔ |  |  |  |  | ✔ |  |  |  |  |  |  | ✔ |
| 2526 | 550 | VI |  |  | ✔ |  |  |  |  |  |  | ✔ |  |  |  |  | ✔ |
| 2533 | 560 | VI |  | ✔ |  |  |  |  |  |  |  | ✔ |  |  |  |  | ✔ |
| 2528 | 560 | VI |  |  |  | ✔ |  |  |  |  |  | ✔ |  |  |  |  | ✔ |
| 2548 | 565 | VI |  |  |  |  | ✔ |  |  |  |  | ✔ |  |  |  |  | ✔ |
| 2530 | 570 | VI |  |  |  | ✔ |  |  |  |  |  | ✔ |  |  |  |  | ✔ |
| 2536 | 570 | VI |  |  |  | ✔ |  |  |  |  |  | ✔ |  |  |  |  | ✔ |
| 2549 | 570 | VI |  |  |  | ✔ |  |  |  |  |  | ✔ |  |  |  |  | ✔ |
| 2546 | 570 | VI |  |  |  |  | ✔ |  |  |  |  | ✔ |  |  |  |  | ✔ |
| 2534 | 590 | VI |  | ✔ |  |  |  |  |  | ✔ |  |  |  |  |  |  | ✔ |
| 2551 | 595 | VI |  | ✔ |  |  |  |  |  |  |  | ✔ |  |  |  |  | ✔ |
| 2527 | 600 | VI |  |  |  |  | ✔ |  |  |  |  | ✔ |  |  |  |  | ✔ |
| 2552 | 605 | VI |  |  |  | ✔ |  |  |  |  |  | ✔ |  |  |  |  | ✔ |
| 2524 | 620 | VI |  |  |  |  | ✔ |  |  |  |  | ✔ |  |  |  |  | ✔ |
| 2531 | 650 | VI |  |  |  |  | ✔ |  |  |  |  | ✔ |  |  |  |  | ✔ |
| 2542 | 660 | VI |  |  |  | ✔ |  |  |  |  |  | ✔ |  |  |  |  | ✔ |
| 2529 | 700 | VI |  |  | ✔ |  |  |  |  |  |  | ✔ |  |  |  |  | ✔ |
| 2539 | 710 | VI |  |  | ✔ |  |  |  |  | ✔ |  |  |  |  |  |  | ✔ |
| 2537 | 730 | VI |  |  |  |  | ✔ |  |  | ✔ |  |  |  |  |  |  | ✔ |
| 2525 | 740 | VI |  |  | ✔ |  |  |  |  | ✔ |  |  |  |  |  |  | ✔ |
| 2532 | 740 | VI |  | ✔ |  |  |  |  |  |  |  | ✔ |  |  |  |  | ✔ |
